# Revisiting ISO 10993‐5 In Vitro Cytotoxicity Standard Tests for Evaluation of Extracellular Matrix‐Based Biomaterials

**DOI:** 10.1155/ijbm/7395612

**Published:** 2026-05-30

**Authors:** Rashmi Ramakrishnan, Andrew C. Daly

**Affiliations:** ^1^ CURAM, Research Ireland Centre for Medical Devices, University of Galway, Galway, H91W2TY, Ireland, universityofgalway.ie; ^2^ Biomedical Engineering, School of Engineering, University of Galway, Galway, H91TK33, Ireland, universityofgalway.ie

**Keywords:** biomaterial, cytocompatibility, cytotoxicity, ECM, in vitro cytotoxicity tests, ISO 10993-5, medical devices, preclinical testing, scaffolds, small intestinal submucosa

## Abstract

Cytotoxicity testing is a critical step in the preclinical evaluation of biomaterials and medical devices, with extracellular matrix (ECM)‐based biomaterials constituting a significant category. The current ISO 10993‐5:2009 standard, Biological Evaluation of Medical Devices‐Part 5: Tests for In Vitro Cytotoxicity, delineates four metabolic assays that primarily rely on colorimetric methods and colony formation. However, relying solely on colorimetric assays or colony formation fails to provide precise insights into cell function/activity and may yield false‐positive results, contributing to interlaboratory discrepancies. This study systematically evaluated ISO 10993‐5‐recommended assays for ECM‐based commercial products, specifically assessing the impact of key assay variables including cell types, contact mode (test extracts versus test material itself), and media components (with or without serum) on biological outcomes. These evaluations support the development of more accurate and robust test methods. While all four assays indicated the noncytotoxic nature of the test samples, metabolic activity readings varied substantially depending on the serum presence, cell types, and assay method employed. To address these limitations and achieve more precise insights into cellular activity, cell membrane integrity (live/dead staining), cell‐ECM attachment (actin cytoskeleton), proliferation (Ki67), and apoptosis (annexin V) were analyzed. Notably, despite observing increased metabolic activity (100%–150%) under serum‐free conditions measured using MTT and XTT assays, live/dead and actin staining showed no corresponding changes in cell viability or attachment, and Ki67 indicated only ∼15% proliferation. Annexin V staining was detected only in human primary dermal fibroblasts, highlighting their greater reliability over L929 cells for detecting apoptosis. These findings provide a valuable reference for researchers, regulatory bodies, and industry stakeholders in refining cytotoxicity testing protocols and guiding future ISO 10993‐5 revisions for more reliable assessment of biomaterials and medical devices.

## 1. Introduction

Biocompatibility assessment is critical in the development and regulatory endorsement of medical devices, assuring their safety and efficacy for human use and for gaining market access [1]. Regulations pertaining to biocompatibility testing and medical devices differ from country to country. Nevertheless, certain internationally recognized standards and regulations are commonly acknowledged and adhered to by the medical device industry. They include the International Organization for Standards (ISO) 10,993 series, Organization for Economic Cooperation and Development (OECD) test guidelines (TGs), American Society for Testing and Materials (ASTM) International, U.S. Food and Drug Administration (FDA), European Medical Device Regulation (MDR), Pharmaceuticals and Medical Devices Agency (PMDA), US Pharmacopeia (USP), Health Canada Regulations guidelines, European Medicines Agency (EMA), etc. Most of these guidelines align with the ISO‐10993 series [1, 2]. Out of which, in vitro cytotoxicity testing as per ISO 10993‐5:2009 is a fundamental testing method mandated by all federal agencies, notified bodies, and regulatory bodies for demonstrating the safety of medical devices [3, 4]. Formulated by the ISO/technical committee (TC) 194, this standard outline recognized in vitro cytotoxicity testing methodologies and protocols for performing the biological assessment of medical devices [2, 3]. These assessments generally involve cultured cells that are either placed in direct contact with a medical device or its extracts or subjected to indirect contacts through techniques such as agar overlay or filter diffusion tests [3, 5]. The sample preparation and reference material selection for medical device testing are executed according to ISO 10993‐12:2021 standard guidelines [6]. The ISO 10993‐5:2009 standard details four specific tests: 3‐[4,5‐dimethylthiazol‐ 2‐yl]‐2,5‐diphenyltetrazolium bromide (MTT), 2,3‐bis‐[2‐methoxy‐4‐nitro‐5‐sulfophenyl]‐5‐carboxanilide‐2H‐tetrazolium (XTT), neutral red uptake (NRU), and colony formation assay (CFA) [3]. The cytotoxicity is assessed by examining cell death or damage, either qualitatively or quantitatively. Qualitative analysis involves microscopic observation of changes in cell morphology, such as alterations in shape, vacuolization, detachment, lysis, and membrane integrity, graded on a scale from 0 to 4. Quantitative analysis measures cell damage/growth through colorimetric assays based on cellular metabolism or CFAs [3, 6–10]. A material is regarded as noncytotoxic when the cells cultured in contact with the test extracts exhibit metabolic activity greater than or equal to (≥) 70% relative to the control group, as per the standard [3, 11].

Despite the widespread use of ISO 10993‐5:2009 test methods, the recommended methodologies within the standard, primarily based on colorimetric assessments of metabolic activity, may not explicitly predict material‐cellular interactions, leading to concerns about effectiveness. Numerous studies have identified critical limitations in these in vitro cytotoxicity assays and emphasized key factors that must be considered when designing study protocols, conducting experiments, analyzing data, and interpreting results [2, 7–19]. Table 1 provides a detailed overview of these test methods, and Figure 1 illustrates the main steps involved in their implementation. For instance, various chemical compounds, including antioxidants, ascorbic acid, tocopherols, dihydrolipoic acid, glutathione, glutathione‐S‐transferase, coenzyme A, cysteine, polyphenolic compounds, superoxides, and cholesterol, have been reported to interfere with cytotoxicity reagents such as MTT and XTT, leading to altered assay outcomes [20, 38–40]. Moreover, extracellular matrix (ECM)‐based scaffolds possess an intricate and complex biochemical composition comprising collagen, elastin, glycosaminoglycans (GAGs), proteoglycans, glycoproteins, fibronectin, tenascin, laminin, integrins, growth factors (GFs), and matrix metalloproteinases, which can interfere with conventional cytotoxicity assessments by adsorbing, sequestering, or neutralizing assay reagents, altering their reactivity and masking toxic effects, thereby reducing the sensitivity of cytotoxicity assays and potentially yielding misleading or inconclusive results ranging from unacceptable to excellent [21, 41–43]. Another difficulty with ECM‐based biomaterials is their batch‐to‐batch inconsistency, which affects reproducibility under ISO 10993‐5 conditions, along with challenges in extract preparation, as ECM scaffolds may degrade or swell inconsistently, making extraction nonstandardized and unpredictable. Other variables influencing assay outcomes include the presence and concentration of serum in the extraction medium, exposure methods, culture conditions, cell type, cell seeding density, cell growth phase, and the nature of cell‐material interactions (such as direct contact versus extract‐based exposure) [9, 10, 13, 14, 16, 18, 19, 41]. Furthermore, prolonged exposure of the assay reagents to light and increased pH in the culture medium may also lead to elevated background absorbance, potentially increasing the likelihood of positive or false result outcomes [12]. Table 2 summarizes studies that critically evaluated the limitations of applying ISO 10993‐5:2009 test methods to various biomaterials. This underscores the challenges in achieving consistent applicability of the ISO 10993‐5 standard.

**TABLE 1:** Comparison of test methods in ISO 10993‐5 in vitro tests for cytotoxicity.

Table 1. ISO 10993‐5 standard in vitro cytotoxicity testing methods							
Tests/assays	Test sample	Cell type, medium, and concentration of FBS	Mechanism, endpoint method, and duration of study	Method criterion and limit value	Advantages	Limitations	References
*Quantitative assays*							
Elution assay	Test material or test extract	*Cell type:* L929; mouse fibroblast cells. *Medium:* DMEM with 2 µM L‐glutamine with 10% FBS.	*Mechanism:* Visual assessment of cell damage by morphological changes and cellular damage measurement. *Endpoint method:* Microscopic examination. *Duration of study*: 24 or 48 h.	The elution test qualitatively assesses cell viability by microscopically examining cells and degree of biological reactivity is assessed using a cytotoxicity grade scale ranging from 0 to 4. A grade above 2 is considered cytotoxic.	(1) Quick turnaround time. (2) Visualization of cellular morphology and intracellular structures. (3) Relatively rapid and less expensive.	(1) More subjective and could be analyst dependent. Considerable variability of results among laboratories. (2) Utilizes cell line rather than the appropriate or relevant cell types. (3) Study duration might be insufficient to correlate to in vivo results.	[3, 6, 7, 10]
Modified MEM elution assay							
Agar overlay/Filter diffusion or indirect contact assay	Test material or test extract	*Cell type:* L929; mouse fibroblast cells. *Medium:* DMEM with 2 µM L‐glutamine with 10% FBS.	*Mechanism:* Agar overlay assays are employed for medical devices with considerable toxicity and bulk filtering properties. In this test, an agar layer is placed over the cells in place of medium and test samples are positioned atop the agar layer for assessment. Filtration methods are suitable for the cytocompatibility evaluation of small molecular weight toxic components. In filter diffusion, cells are seeded on the filter surface and positioned on a solidified agar layer that contains the culture medium, with the cell side oriented downward. Subsequently, the specimens of the sample material are positioned on the upper side of the filter for assessment. *Endpoint method:* Microscopic examination. *Duration of study:* Agar overlay (24 h) & Filter diffusion (2.0 ± 0.1 h).	The degree of biological reactivity is assessed using a cytotoxicity grade scale ranging from 0 to 4. A grade above 2 is considered cytotoxic.	(1) Quick turnaround time. (2) Relatively rapid and less expensive.	(1) Cells may not be entirely exposed to the test material, if the potential cytotoxic leachates cannot diffuse across the agar. (2) Released compounds from the biomaterial/medical device can react with the agar. (3) Released constituents from the test material can bind to the filter. (4) Biomaterial exposure or testing time might be insufficient to correlate to in vivo results. (5) Utilizes cell line rather than the appropriate or relevant cell types. (6) Constituents of media can interfere with the test. (7) Agar thickness and serum concentration can interfere with the test results.	[3, 5, 7, 10]
Direct contact	Test material or test extract	*Cell type:* L929; mouse fibroblast cell. *Medium:* DMEM with 2 µM L‐glutamine with 10% FBS.	*Mechanism:* Visual assessment of cell damage by morphological changes and cellular damage measurement. *Endpoint method:* Microscopic examination. *Duration of study:* 24 or 48 h.	The degree of biological reactivity is assessed using a cytotoxicity grade scale ranging from 0 to 4. A grade above 2 is considered cytotoxic.	(1) Quick turnaround time. (2) Visualization of cellular morphology and intracellular structures. (3) Relatively rapid and less expensive.	(1) Cells in direct contact with a test substance may be more susceptible to mechanical stress, particularly from the applied weight, increasing the likelihood of physical damage. (2) Utilizes cell line rather than the appropriate or relevant cell types. (3) Study duration might be insufficient to correlate to in vivo results. (4) Considerable variability of results among laboratories.	[3, 6, 7, 10, 14]

*Quantitative assays*							
MTT assay	Test extract	*Cell type*: L929; mouse fibroblast cell. *Medium:* DMEM with 2 µM L‐glutamine with 10% FBS. *Mechanism:* Intracellular reduction of yellow, water‐soluble MTT occurs via the mitochondrial (NAD(P)H) coenzyme and dehydrogenases, leading to the formation of blue‐violet or purple insoluble formazan, which is subsequently solubilized, and absorbance is measured. *Endpoint method:* Absorbance measurement. *Duration of study:* 24 h. 		Cell viability is expressed on a scale of 0%–100%. If there is reduction of cell viability of 30% or above, the material is considered cytotoxic.	(1) Potential for developing dose‐response curves. (2) Relatively rapid and less expensive.	(1) MTT as such, is cytotoxic to cells. (2) MTT needs additional solubilization step prior to absorbance measurement and based on solvents used such as isopropyl alcohol, dimethyl sulfoxide (DMSO), or dimethylformamide (DMF), etc, can affect reproducibility. (3) Endpoint measurement only and limited information on morphological changes. (4) Test cannot distinguish between cytotoxic and cytostatic effects. (5) Some test substances (e.g., coloured or reducing agents, serum) can interfere with MTT readouts. (6) Utilizes cell line rather than the appropriate or relevant cell types. (7) Considerable variability of results among laboratories. (8) Test may not identify effects that develop gradually over time or due to the accumulation of substances.	[3, 6, 8, 10–15, 20–28]
XTT assay	Test extract	*Cells:* L929; mouse fibroblast cell. *Medium:* DMEM with 2 µM L‐glutamine with 10% FBS. *Mechanism:* Extracellular reduction of yellow XTT by NADH generated in the mitochondria into orange formazan dye in aqueous solutions and its absorbance is measured. *Endpoint method:* Absorbance measurement. *Duration of study:* 24 h. 		Cell viability is presented in the range of 0%–100%. If there is reduction of cell viability of 30% or above, the material is considered cytotoxic.	(1) Potential for developing dose‐response curves. (2) Relatively rapid and less expensive.	(1) Sensitivity of the assay varies. (2) Strongly influenced by cellular metabolic activity, specifically dehydrogenase enzymes, assay results can be significantly altered by various in vitro conditions like cell growth phase, population density, nutrient availability, pH, and other factors affecting cellular reductive capacity. (3) Some test substances (e.g., coloured or reducing agents, serum etc) can interfere with XTT readouts. (4) The XTT reagent is more prone to light sensitivity and degradation, requiring careful storage and handling. (5) Utilizes cell line rather than the appropriate or relevant cell types. (6) Considerable variability of results among laboratories. (7) Limited information on morphological changes.	[3, 6–10, 12–14, 20, 26, 27, 29]
NRU assay	Test extract	*Cells:* BALB/c 3T3; mouse embryonic fibroblast cells. *Medium:* DMEM with 2 µM L‐glutamine with 5% FBS + 5% newborn calf serum (NBCS). *Mechanism:* The capacity of viable and uninjured cells to uptake and bind the neutral red dye to the functional lysosomes is measured by reading absorbance of the solubilized dye. *Endpoint method:* Absorbance measurement. *Duration of study:* 24 h. 		Cell viability is presented in the range of 0%–100%. If there is reduction of cell viability of 30% or above, the material is considered cytotoxic	(1) Potential for developing dose‐response curves. (2) Nontoxic to cells. (3) Relatively rapid and less expensive.	(1) Test substances causing lysosomal swelling may underestimate toxicity. Neutral red dye interference may lead to high background. (2) Damaged or stressed cells may release the dye prematurely, leading to underestimation of viability. (3) Considerable variability of results among laboratories. (4) Test may not identify effects that develop gradually over time or due to the accumulation of substance. (5) Serum and other components from biomaterials can react with the reagents causing interference in the results. (6) Limited information on morphological changes. (7) Utilizes cell line rather than the appropriate or relevant cell types.	[3, 6, 8, 11, 14, 15, 25, 26, 30–32]
CFA or clonogenic assay	Test extract	*Cell type:* V79‐4 cells; hamster. *Medium:* DMEM with 2 µM L‐glutamine with 10% FBS (direct) or 5% FBS (extract). *Mechanism:* The potential of a cell to multiply and develop into a population/colony after cytotoxic agent administration for a prolonged period of 6 days is measured using a stereo microscope after staining. *Endpoint method:* Microscopic examination. *Duration of study:* 6‐7 days.		Reduction of relative plating efficiency of more than 30% at 100% concentration of the test extract is considered as a cytotoxic effect.	(1) Measures long term survival. (2) Relatively less expensive.	(1) Considerable variability of results among laboratories. More subjective and could be analyst dependent. (2) Restricted to adherent cells; moreover, not all adherent cells can establish colonies in vitro at low cell density where cell‐to‐cell interactions and self‐produced growth factors are scarce. (3) Utilizes cell line rather than the appropriate or relevant cell types. (4) Laborious and time‐consuming.	[3, 6, 33–37]

*Note:* All pictorial illustrations are created with BioRender.com/BioRender‐templates.

Abbreviations: MTT (3‐[4,5‐dimethylthiazol‐2‐yl]‐2,5‐diphenyltetrazolium bromide), XTT (2,3‐bis‐[2‐methoxy‐4‐nitro‐5‐sulfophenyl]‐5‐carboxanilide‐2H‐tetrazolium), NRU (neutral red uptake), and CFA (colony formation assay).

FIGURE 1Schematic representation showing: (a) in vitro cytotoxicity test methods for cytotoxicity assessment of biomaterials like test on extracts, direct contact test, indirect contact agar overlay/diffusion test, and indirect contact filter diffusion test, (b) possible cell response after biomaterial contact, and (c) detailed protocol of ISO 10993:5 recommended tests—MTT (3‐[4,5‐dimethylthiazol‐2‐yl]‐2,5‐diphenyltetrazolium bromide), XTT (2,3‐bis‐[2‐methoxy‐4‐nitro‐5‐sulfophenyl]‐5‐carboxanilide‐2H‐tetrazolium), neutral red uptake (NRU), and colony formation assay (CFA). All pictorial representations are created with BioRender.com.

**Figure:**
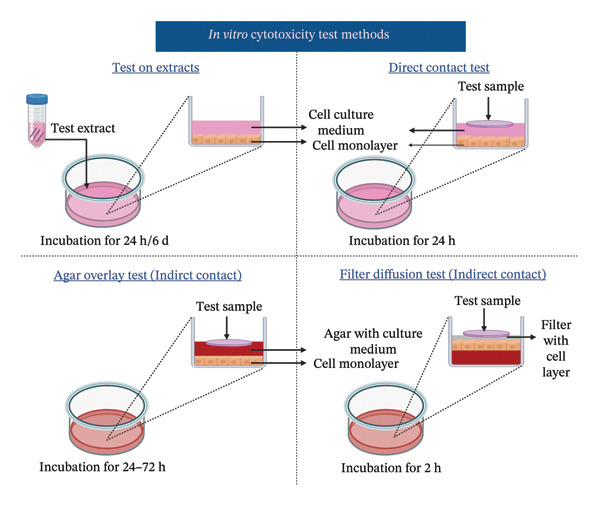
(a)

**Figure:**
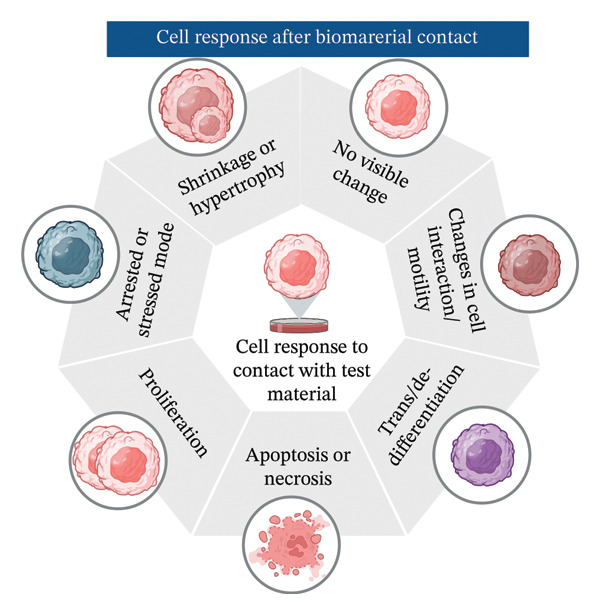
(b)

**Figure:**
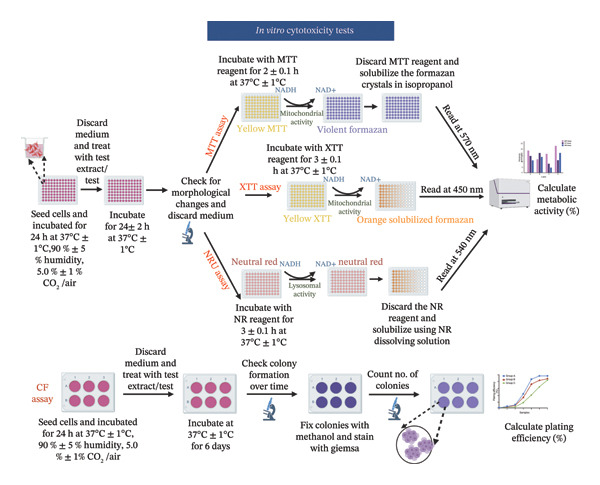
(c)

**TABLE 2:** Overview of studies highlighting the challenges of implementing ISO 10993‐5 standard.

Table 2. Challenges in application of ISO 10993‐5 standard		
Key complications in the ISO 10993‐5 standard	Key insights of the study	References
Inadequate for certain biomaterials	• Calcium (amorphous, α‐tricalcium, hydroxyapatite, and octacalcium) phosphate‐ based biomaterials were tested using ISO 10993‐5 standard. • The contact type exhibited variability in test outcomes, with extract‐based assays failing to detect cytotoxicity, whereas direct contact tests at physiologically relevant concentrations revealed reduced metabolic activity. • The impact of material form (especially powders), interaction dynamics, and realistic cell models for accurate cytocompatibility assessments were highlighted.	[11]
	• The inaccuracy of XTT or MTT test methods for assessing cell viability due to nano‐TiO_2_ induced superoxide formation resulting in over or under estimated cell toxicity or viability were highlighted.	[20, 44]

Interlaboratory variability	• The cytotoxicity of polyethylene and polyvinyl chloride tubing samples (food grade, ethylene oxide sterilised) over 52 international laboratories were compared. • Only 58% of the tested laboratories correctly identified the cytotoxic potential of the tested materials. • Serum concentration, extraction conditions and incubation time were found to influence test sensitivity.	[9]

Sensitivity to test conditions and need for material‐specific protocols	• The in vitro cytotoxicity test results of degradable metallic (zinc‐based and magnesium‐based) biomaterials were shown to be varied with test conditions. • Extraction conditions including medium type, serum concentration, exposition mode, and cell type were shown to have impact on the cytotoxicity of the extracts.	[13]
	• The variability in cell line, medium, and controls were shown to significantly affect the outcomes when testing antimicrobial electrospun biomaterials.	[45]
	• The in vitro cytotoxicity test conditions for biodegradable magnesium‐based materials were studied. • The differences in cell sensitivities to test dilutions including ion concentration, pH and osmolality were discussed.	[17]
	• The variability of in vitro cytotoxicity test results of perfluorocarbons intended for intraocular use was compared using cell lines and ex vivo human donor retina studies. • This study validates that only specific test conditions under ISO 10993‐5 could reveal cytotoxicity for certain materials, showing the importance of detailed test setup reporting.	[46]
	• The impact of culture conditions and specimen size on cytotoxicity outcomes for oral biomaterials (resin composites) were tested, demonstrating that even slight deviations from ISO 10993‐5 can significantly affect results.	[47]

Material contact‐type variation	• The variability between direct contact and extract exposure methods for perfluorocarbon (PFO) cytotoxicity evaluation were investigated. Direct contact testing revealed significantly higher cytotoxicity of PFO compared to extract exposure. • They also found that compared to short‐term (30 min) cell exposure to the test solution, an extended (72 h) postexposure growth period masked potential cytotoxic effects, during which surviving cells proliferated and compensated for earlier cellular damage.	[19]

Herein, this study systematically evaluates ISO 10993‐5‐recommended in vitro cytotoxicity assays in the context of ECM‐based biomaterials, focusing on key variables such as cell type, contact type (test extracts or test material itself), and media components (±serum) on cell viability outcomes using commercially available ECM‐based products. While ECM biomaterials are known for their cytocompatibility in several clinical applications, they can exhibit batch‐to‐batch variability in terms of chemical, biological, and physical properties. The chemical composition, including tissue source characteristics, ECM composition, bioactive molecules, decellularization method (physical, chemical, or enzymatic), decellularization efficiency, cross‐linking agents, residual chemicals, sterilization methods, storage conditions, and degradation products, can all impact cytotoxicity and modulate cell behaviors such as adhesion, differentiation, and migration [29, 48]. Additionally, batch‐to‐batch variability in physical properties such as porosity, mechanical properties, and degradation rates can impact cell‐material interactions and therefore cytotoxicity assay readouts [15, 42, 43, 49, 50]. In this study, an ECM‐based scaffold, Biodesign1‐Layer Tissue Graft from Cook Biotech (now Evergen, USA), made from naturally occurring ECM obtained from the small intestinal submucosa (SIS), was primarily evaluated. This scaffold is used in clinics to reinforce soft tissue and in applications such as surgical hernia repair, fistula repair, and otologic repair [51]. Another ECM‐based product, Endoform Natural Restorative Bioscaffold from Aroa Biosurgery Ltd. (New Zealand), was also tested as a comparison. The Endoform scaffold is based on ruminant forestomach ECM and is used for the management of acute and chronic wounds [52, 53]. Both scaffolds were obtained terminally sterilized via ethylene oxide and intended for single use. Initially, the impact of the presence or absence of serum ( ± fetal bovine serum [FBS]) on cytotoxicity measurements of ECM‐based biomaterials was evaluated using ISO 10993‐5‐recommended tests. Following this, cell‐type‐specific responses to the ECM‐based biomaterials were evaluated by performing the recommended tests using several cell types outlined in ISO 10993‐5. Finally, the cytocompatibility was accessed using live/dead staining, cytoskeletal actin immunostaining, proliferation assessment via Ki67 immunostaining, and apoptosis assessment using annexin V staining, which are not currently included in the ISO 10993‐5 standard. By directly comparing these methods with the recommended colorimetric assays, the study aimed to determine if the ISO 10993‐5 standard requires amendment. These alternative methods may offer a quicker and easier means to gain insights into cell functionality compared to the conventional colorimetric assay, while providing a more profound and comprehensive assessment of cellular responses to biomaterials.

## 2. Materials and Methods

### 2.1. Cell Culture and Consumables

L929 (NCTC clone 929 cells; catalog no.: 85,011,425), BALB/c 3T3 (BALB/c 3T3 clone A31 cells; catalog no.: 86,110,401) and V79‐4 cells (catalog no.: 93,010,723) were purchased from Sigma, USA, and were used for the study. Primary normal human dermal fibroblasts (HDF) were purchased from PromoCell GmBH, Germany (catalog no.: C‐12302), and the human colon epithelial Caco‐2 cell line was purchased from American Type Culture Collection, USA (ATCC; catalog no.: HTB‐37). The cells were culture‐expanded as per the supplier’s instruction and recommended culture media. Reagents like MTT (catalog no.: M2128, Sigma‐Aldrich, USA), XTT cell proliferation kit II (catalog no.: 11,465,015,001, Roche, Switzerland), NR cell viability/cytotoxicity assay kit (catalog no.: ab 234,039, Abcam, USA), Giemsa stain (catalog no.: G5637, Sigma‐Aldrich, USA), isopropanol (catalog no.: I9516; Sigma‐Aldrich, USA), methanol (catalog no.: 34,860; Sigma‐Aldrich, USA), sodium dodecyl sulfate (SDS; catalog no.: L3771; Sigma‐Aldrich, USA)/sodium lauryl sulfate (SLS; catalog no.: S529, Fisher Scientific, USA), bovine serum albumin (BSA; catalog no.: A7638; Sigma‐Aldrich, USA), triton‐X‐100 (catalog no.: 93,443; Sigma‐Aldrich, USA), paraformaldehyde (catalog no.: P6148; Sigma‐Aldrich, USA), calcein AM (catalog no.: 56,496, Sigma‐Aldrich, USA), ethidium homodimer‐1 (EthD‐1; catalog no.: E1903, Sigma‐Aldrich, USA), rhodamine phalloidin reagent (catalog no.: ab235138, Abcam, USA), 4′,6‐diamidino‐2‐phenylindole dihydrochloride (DAPI; catalog no.: D1306, Invitrogen, Thermo Fisher scientific, USA) or Hoechst 33,342 (catalog no.: 62,249, Thermo Scientific, USA), Ki67 (catalog no.: ab16667, Abcam, USA), secondary antibody (goat antirabbit IgG H&L (Alexa Fluor [AF] 488, catalog no.: ab150077, Abcam, USA) and annexin‐V‐PE apoptosis staining kit (catalog no.: ab14155, Abcam, USA) were procured from the corresponding manufacturer enclosed in brackets next to the chemical.

### 2.2. Sample Preparation

The test samples used were a porcine SIS‐based scaffold (Biodesign 1‐Layer Tissue Graft, Cook Biotech, now Evergen, USA) and a ruminant forestomach ECM‐based scaffold (Endoform Natural Restorative Bioscaffold, Aroa Biosurgery Ltd., New Zealand), termed SIS1 and EF, respectively. Both the scaffolds were obtained sterile. The test samples were compared to a toxic positive control (PC; 0.1 mg/mL sodium dodecyl sulfate or sodium lauryl sulfate; SDS/SLS) and cells‐only control (i.e., cells growing on TCPS without [W/O] any treatment). The study also assessed the influence of serum (FBS) in test and culture medium conditions. For test‐on‐extract tests, samples were extracted in accordance with the ISO 10993‐12:2021 standard, with a recommended extraction ratio of 6 cm^2^ of surface area per mL of extraction volume, or 0.1 g of sample mass per mL of extraction volume. In brief, the test samples were incubated with and without (W/O) FBS (± FBS)‐containing culture medium for 24 ± 2 h at 37°C ± 1°C with gentle agitation at 100 rpm to extract out the potential leachates. After incubation, the test extracts were separated by decantation, designated as 100% extract. Subsequently, the 100% extract was diluted using ± FBS‐containing culture medium to give various percentages of diluted extract, such as 50%, 25%, and 12.5%. For the Endoform and cell controls, 100% test extract or 100% relevant cell culture medium ± FBS was used, respectively. The extracts were then fed to monolayer cell cultures for 24 ± 2 h at 37°C ± 1°C in CO_2_ incubator. Cell types like L929, BALB/c 3T3, and V79‐4 were used for direct contact, MTT, XTT, NRU, and CFA, accordingly, as per the 10,993‐5 standard. HDF and Caco‐2 cells were also used for the evaluation as a comparison. For direct contact tests, test samples were punched into desired sizes aseptically and were either placed directly in contact with the L929 cells or cells were seeded directly onto the test material. Postincubation, cell cytotoxicity was assessed by analyzing: (i) cell morphology (grading on a scale of 0–4 as per ISO 10993‐5 standard), (ii) ISO 10993‐5 recommended metabolic assays like MTT, XTT, NRU, and CFA, and (iii) staining methods like live/dead assay, cytoskeletal actin, and proliferation marker‐Ki67 immunocytochemistry as well as apoptotic marker‐annexin V staining. Staining methods were performed using both L929 and HDF cells.

### 2.3. ISO 10993‐5 Recommended Assays

#### 2.3.1. MTT Assay

The cytotoxicity of the test material was studied using an MTT assay by comparing cell metabolic activity of L929, HDF, and Caco‐2 cells, in compliance with the ISO 10993‐5 standard. Briefly, 10,000 cells per well of a 96‐well tissue culture microtiter plate with appropriate cell culture medium ± FBS with at least three replicates per condition were cultivated for 24 h ± 2 h at 37°C ± 1°C in a CO_2_ incubator in a humidified environment (90% ± 5% humidity, 5% ± 1% CO_2_/air). After 24 h, uniform cell growth was confirmed using an inverted microscope to identify experimental errors, and the cells were treated with either test extracts or control medium under the same condition for another 24 h ± 2 h. Four varying test extract concentrations of SIS1 as 100%, 50%, 25%, and 12.5% and a 100% test extract of Endoform were used for the study. For the cell controls, relevant cell culture medium ± FBS was added, respectively. Postincubation, the plates were inspected for any morphological changes, and the treatment/control medium was discarded, followed by gentle washing with prewarmed PBS (pH 7.4). Later, freshly prepared MTT solution (1 mg/mL, sterile filtered) was added and incubated for 2.0 h ± 0.1 h in the dark at 37°C under the same condition for the conversion of yellow MTT into water‐insoluble purple formazan products by the metabolically active cells. Following incubation, the resulting formazan crystals were dissolved using isopropanol with gentle swaying after decanting the MTT solution. The resultant solution was used for measuring absorbance employing a microplate reader (Varioskan Flash spectral scanning multimode reader with SkanIt software‐4.00.53, Thermo Fisher Scientific, Finland) equipped with a 570 nm filter. The cell metabolic activity or viability was assessed using the given equation (1):

(1) Cell viability %=OD570test−OD570blankOD570cell control−OD570blank×100,

where OD_570test_ is the test optical density/absorbance measured, OD_570blank_ is the blank optical density/absorbance measured, and OD_570cell control_ is the cell control optical density/absorbance measured.

#### 2.3.2. XTT Assay

The cytotoxicity of test material was evaluated using the XTT assay by comparing cell metabolic activity of L929, HDF, and Caco‐2 cells, in accordance with the ISO 10993‐5 standard. Briefly, cells were seeded, and treatment was done as mentioned in Section 2.3.1, and the XTT assay was carried out according to the manufacturer’s instructions. Posttreatment, the plates were inspected for morphological changes under a microscope. Later, the treatment/control medium was removed, gently washed with prewarmed PBS, and freshly prepared XTT/PMS solution was then added to each well and subjected to 3.0 ± 0.1 h incubation in the dark at 37°C in a CO_2_ incubator for the metabolically active cells to convert the yellow XTT reagent to water‐soluble orange formazan products. The solution’s absorbance was read employing a microplate reader (Varioskan Flash spectral scanning multimode reader with SkanIt software‐4.00.53, Thermo Fisher Scientific, Finland) equipped with a 450 nm filter. The cell metabolic activity or viability was assessed employing the following equation (2):

(2) Cell viability %=OD450test−OD450blankOD450cell control−OD450blank×100,

where OD_450test_ is the test optical density/absorbance measured, OD_450blank_ is the blank optical density/absorbance measured, and OD_450cell control_ is the cell control optical density/absorbance measured.

#### 2.3.3. NRU Assay

The cytotoxicity of test material was evaluated using the NRU assay by comparing cell metabolic activity of L929, HDF, and Caco‐2 cells, in compliance with the ISO 10993‐5 standard. Briefly, cells were seeded, and treatment was done as mentioned in Section 2.3.1, and the NRU assay was carried out according to the manufacturer’s instructions. Posttreatment time, the cells were examined for any morphological changes under a microscope. The culture medium of the test/control was replaced, gently washed with prewarmed PBS, treated with the NR reagent medium, and incubated in the dark for 3.0 h ± 0.1 h in a CO_2_ incubator. Postincubation, NR desorption solution was used to extract the NR deposited inside the cells with gentle swaying for 10 min in the dark. The solution was measured for its absorbance at 540 nm ± 10 nm with the aid of a microplate reader (Varioskan Flash spectral scanning multimode reader with SkanIt software‐4.00.53, Thermo Fisher Scientific, Finland). The cell viability or metabolic activity was then calculated using the following equation (3):

(3) Cell viability %=OD540test−OD540blankOD540cell control−OD540blank×100,

where OD_540test_ is the test optical density/absorbance measured, OD_540blank_ is the blank optical density/absorbance measured, and OD_540cell control_ is the cell control optical density/absorbance measured.

#### 2.3.4. CFA

The cytotoxicity of test material using the colony formation or clonogenic cytotoxicity test was assessed using V79‐4 cells. Briefly, 100 cells per well in a 6‐well tissue culture plate were cultivated in ± FBS cell culture medium for 24 ± 2 h at 37°C ± 1°C in a CO_2_ incubator in a standard humidified atmosphere (90% ± 5% humidity, 5% ± 1% CO_2_/air). Postincubation, the culture medium was drawn out from each well and test extracts (100% extracts) were added in triplicates and incubated for 6 days to form colonies large enough to count. After 6 days, the treatment medium was removed, cell colonies were gently rinsed with prewarmed PBS, fixed with methanol, and stained with 5% Giemsa solution. The total number of colonies having 50 or more cells in each well was manually estimated under a stereo zoom microscope (Leica MZ6, USA), and the average colony number for tests/controls were calculated. The quotient (plating efficiency [PE]) was calculated using equation (4):

(4) Plating  efficiency %=Number of colonies formed in testNumber of colonies formed in control×100.

### 2.4. Direct Contact Assay

A direct contact assay was conducted using L929 cells, in compliance with the ISO 10993‐5 standard. Briefly, 20,000 L929 cells per well of a 48‐well tissue culture microtiter plate were seeded and cultured for 24 h to reach sub‐confluency. The normal cell morphology and uniform growth were verified using an inverted microscope, and the culture medium was then replaced with either fresh medium or different dilutions of test extracts, depending on the biomaterial contact type (test‐on‐extract/test) and incubated for 24 h. For test‐on‐extract, four varying test extract concentrations of SIS1 as 100%, 50%, 25%, and 12.5% and 100% test extract of Endoform were evaluated. For direct test sample contact, individual test specimens were placed in the center of each vessel, covering approximately 1/10th of the cell layer surface. After incubation, changes in cell morphology, including vacuolization, detachment, cell lysis, and membrane integrity, were assessed microscopically, compared with cell control and controls (noncytotoxic negative control (NC; SpongeCol) and toxic PC (0.1 mg/mL SDS/SLS), and documented descriptively or numerically on a scale of grade 0‐4 [3, 5]. The cytotoxicity of the test material based on biomaterial contact type (test or test extract) was further evaluated using the MTT assay for 100% test extract and test material by comparing the cell metabolic activity of L929, HDF, and Caco‐2 cells as mentioned in 2.3.1. For the MTT assay, 100% test extract was added to the seeded cells in the test‐on‐extract group, while in the test‐material group, cells were seeded directly onto the test surface.

### 2.5. Staining Methods

#### 2.5.1. Live/Dead Assay

The live and dead cells on the test sample were distinguished by employing live/dead staining. Briefly, the L929 and HDF cells were seeded directly on test material as well as with test extract (100% extract) and cultured for 24 h and 72 h with ± FBS‐containing cell culture medium, respectively. After the incubation period, the medium was discarded, gently rinsed with prewarmed PBS, and treated with a live‐dead dye solution comprising 2 μM calcein AM and 4 μM ethidium homodimer‐1 (EthD‐1) in serum‐free media for 15–20 min in dark in a CO_2_ incubator. Following incubation, the dye solution was discarded, washed with prewarmed PBS, and immediately examined under an inverted fluorescent microscope (Olympus IX81, Japan) or the operetta high‐content imaging system, utilizing harmony high‐content imaging and analysis software (PerkinElmer Inc., USA) with an excitation and emission (Ex/Em) at ∼495 nm/∼515 nm and ∼495 nm/∼635 nm for calcein AM and EthD‐1, respectively, and compared with cell control. For fluorescence measurements, fluorescence images were converted into separate channels (green and red channels), changed to 8 bit images, and thresholded to separate the signal from the background, and live/dead cells were estimated with the help of FIJI open‐source image analysis software (NIH, USA). The total cell number and the total live and dead cells percentages were then calculated using the given equations (5)–(7), respectively.

(5) Total cell number=Total number of live cells+Total number of dead cells,

(6) Live cells %=Total number of live cellsTotal cell number×100,

(7) Dead cells %=Total number of dead cellsTotal cell number×100.

#### 2.5.2. Actin Cytoskeletal Staining

The cytoskeleton of the cells was stained using actin phalloidin to visualise cell adhesion and distribution on the test samples. The L929 and HDF cells were seeded directly on the test sample as well as on test extracts (100% extract) and cultured for 24 h and 72 h with ± FBS‐containing cell culture medium, respectively. After culture time points, the treatment/control medium was removed, gently rinsed with PBS, and the samples were fixed using 3.7% formaldehyde or 4% paraformaldehyde in PBS for 15 min at room temperature and gently rinsed with PBS (2‐3 times). Later, the samples were permeabilized using 0.1% triton‐X‐100 solution for 5 min at room temperature and washed with PBS (2‐3 times). To reduce nonspecific background staining, samples were blocked using 0.1% BSA in PBS for 30 min at room temperature and washed with PBS (2‐3 times). The actin filaments in the samples were stained with rhodamine phalloidin actin (1:1000) solution at room temperature for 45 min in the dark and washed with PBS (2‐3 times), and DAPI/Hoechst 33,342 (1:1000) was counter‐stained in the dark for 5 min at room temperature to visualize cell nuclei and washed gently with PBS (2‐3 times). The images were captured with the help of operetta high‐content imaging system, utilizing harmony high‐content imaging and analysis software (PerkinElmer Inc., USA) with an Ex/Em at ∼546 nm/∼575 nm and compared with cell control. Quantification of fluorescence was done using FIJI open‐source image analysis software (NIH, USA) by estimating corrected total cell fluorescence (CTCF) using the following equation (8). Background areas were utilized to correct autofluorescence.

(8) CTCF=Integrated density−Area of select×Mean fluorescence of background readings.

#### 2.5.3. Ki67 Staining

Cell proliferation was analyzed using Ki67 immunostaining. The L929 and HDF cells were seeded directly on the test sample and with test extract (100% extract) for 24 h and 72 h with ± FBS‐containing cell culture medium, respectively. After the culture time points, the samples were washed, fixed, permeabilized, blocked, and washed as mentioned in Section 2.5.2. The samples were then stained for primary antibody Ki67 (1:250) overnight at 4°C, washed with PBS (2‐3 times) and stained with secondary antibody AF 488 (1:1000) for 3 h in the dark at room temperature, followed by gentle washing with PBS (2‐3 times). The cell nuclei were counter‐stained using DAPI (1:1000) for 5 min at room temperature and washed with PBS (2‐3 times). Then, the samples were analyzed under operetta high‐content imaging system, utilizing harmony high‐content imaging and analysis software (PerkinElmer Inc., USA) with an Ex/Em at ∼495 nm/∼519 nm and compared with cell control. For measurements of Ki67 positivity, fluorescence images were split into channels (nuclei blue and Ki67 green channels), converted to 8 bit images and thresholded to separate the signal from the background using FIJI open‐source image analysis software (NIH, USA). The total number of cells was calculated by quantifying DAPI‐positive cells, and the % of Ki67‐positive cells was calculated using equation (9).

(9) Percentage of Ki67100+cells %=Total number of Ki67+cellsTotal cell number×.

#### 2.5.4. Annexin V Staining

Cell apoptosis was analyzed using apoptotic marker annexin V‐conjugated with phycoerythrin (PE) staining. Briefly, L929 and HDF cells were seeded on the test material directly as well as on the test extract (100% extract) and cultured for 24 h with ± FBS‐containing cell culture medium, respectively. After terminating the study, annexin V binding buffer was used to wash the cells and incubated in annexin V‐PE staining solution for 5–10 min at room temperature in the dark, as per the manufacturer’s instruction. The samples were then fixed in 4% paraformaldehyde, and the cell nuclei were counter stained using Hoechst 33,342. The images were obtained through the operetta high‐content imaging system, utilizing harmony high‐content imaging and analysis software (PerkinElmer Inc., USA) with an Ex/Em at ∼546 nm/∼575 nm and compared with cell control. Hoechst‐positive cells were quantified to estimate the total number of cells, and the annexin V‐PE‐positive cells % was calculated using equation (10).

(10) Percentage of Annexin V−PE+cells %=Total number of Annexin V−PE+cellsTotal cell number×100.

### 2.6. Statistical Analysis

Data are compiled in a Microsoft Excel file, and GraphPad Prism (version 10) software was used to carry out all the statistical analyses, ensuring a sample size of at least three for each experimental group. All numerical data are presented as mean ± standard deviation (S.D.), with the exact sample number (*n*) of biologically independent replicates indicated in the corresponding figure legends. One‐way analyses of variance (ANOVA) with Bonferroni posttests and two‐way ANOVA with Turkey’s posttests were employed to compare between groups. A *p* value of < 0.05 (^∗^ *p* < 0.05) or less (^∗∗^ *p* < 0.01, ^∗∗∗^ *p* < 0.001, and ^∗∗∗∗^ *p* < 0.0001) is considered as statistically significant difference, as indicated in figure legends, respectively.

## 3. Results and Discussion

### 3.1. Influence of ISO 10993:5 Recommended Metabolic Assays on Cytotoxicity Assessment of ECM Biomaterials

First, the study compared ISO‐recommended assays to examine potential variations in assessing the cytotoxicity of ECM‐based biomaterials. Figure 1 illustrates the test methodologies in detail. L929 cells were used for conducting MTT and XTT assays, BALB/c 3 T3 cells for the NRU assay, and V79‐4 cells for the CFA, as per the ISO 10993‐5 standard. The % metabolic activity of test extracts at different dilutions was compared with untreated cell controls (i.e., cells growing on TCPS without any treatment) and toxic positive controls (PC; 0.1 mg/mL SDS/SLS). All assays were performed with and without FBS ( ± FBS) in the test extraction and culture media. The % of cell metabolic activity was calculated by normalizing the optical density with that of the untreated cell control. A lower viability percentage indicates a higher cytotoxic potential of the test item. A test meets the quality acceptance criteria when the mean O.D. value of blanks is ≥ 0.3 and if the mean O.D. value of the left and right sides of the blanks do not differ by more than 15% from the mean of all blanks [3]. The PC showed an expected cytotoxic response with % metabolic activity below 70% in ± FBS culture groups in all the assays (Figures 2(a) and 2(b)). The test extracts from the SIS1 scaffolds were noncytotoxic at all dilutions, with % metabolic activity above 70% across all assays in both ± FBS culture groups (Figures 2(a) and 2(b)). Similarly, the 100% test extract of Endoform showed metabolic activity above 70% for all assays (Figures 2(a) and 2(b)). Interestingly, the SIS1 extracts at different dilutions showed significantly increased metabolic activity when FBS was not present in the test extraction and culture conditions for both the MTT and XTT assays (Figure 2(b)). This effect was not observed in the NRU assay and also for the Endoform scaffold extracts (Figure 2(b)). In the CFA, the mean number of colonies formed in the cultures is indicative of cell survival rates after test treatment, and the PE % can be estimated by normalising the total colony count to untreated cell controls. The number of colonies was higher in FBS‐containing test extraction and culture conditions. The PEs of 100% test extracts from both ECM‐based scaffolds were above 70% in ± FBS conditions when compared to cell controls, further confirming the noncytotoxic nature of the scaffolds (Figures 2(c) and 2(d)).

FIGURE 2Evaluation of cytotoxicity or metabolic activity using: (a and b) quantitative analysis of MTT, XTT, and NRU cytotoxicity assays after 24 h of culture with test extracts (different dilutions of SIS1 test extracts—100%, 50%, 25%, and 12.5% and Endoform [EF]—100%) compared between controls (untreated cell control growing on TCPS and positive control (PC; diluted SDS/SLS) and serum‐containing (with FBS) and serum‐free (W/O FBS) culture and extraction condition groups, respectively. Bar graphs represent *n* = 4 biological replicates, mean ± S.D.; ^∗∗^ *p* < 0.01, ^∗∗∗^ *p* < 0.001 and ^∗∗∗∗^ *p* < 0.0001 versus cell control, and (c and d) representative photographic images showing qualitative and quantitative analysis of colony formation after 6 days of culture with 100% test extracts of SIS1 and EF compared between controls (untreated cell control and PC; diluted SDS/SLS) and ± serum (with FBS or W/O FBS) in cell culture and extraction conditions groups, respectively. Bar graphs represent *n* = 3 biological replicates, mean ± S.D.; ^∗^ *p* < 0.05, ^∗∗^ *p* < 0.01, ^∗∗∗^ *p* < 0.001, and ^*∗∗∗∗*^ *p* < 0.0001 versus cell control and  ± serum groups.

**Figure:**
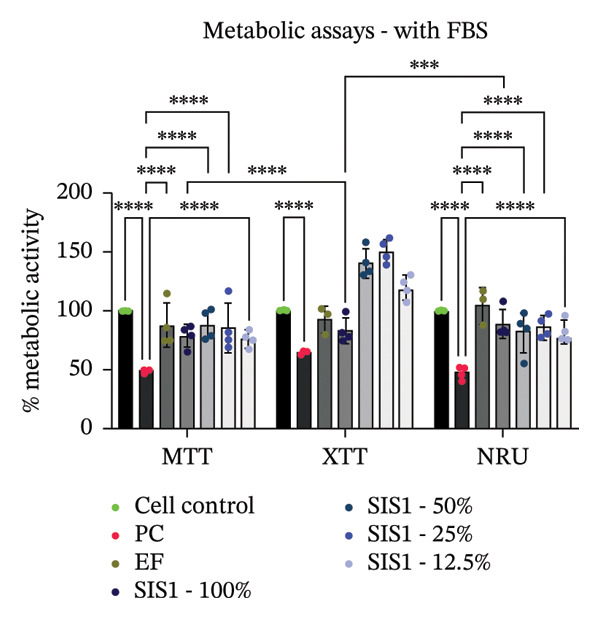
(a)

**Figure:**
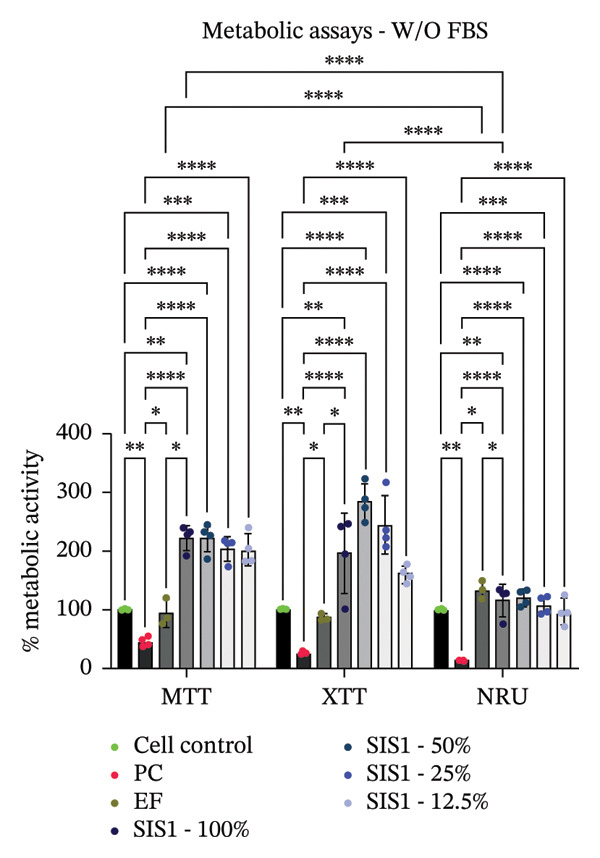
(b)

**Figure:**
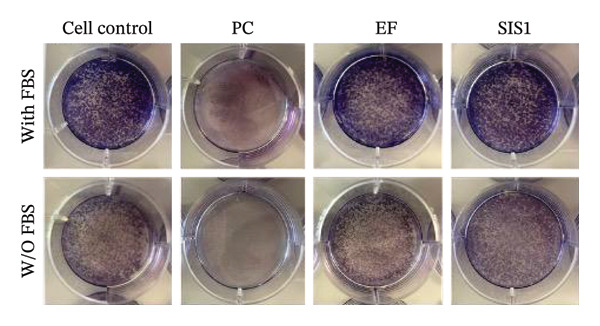
(c)

**Figure:**
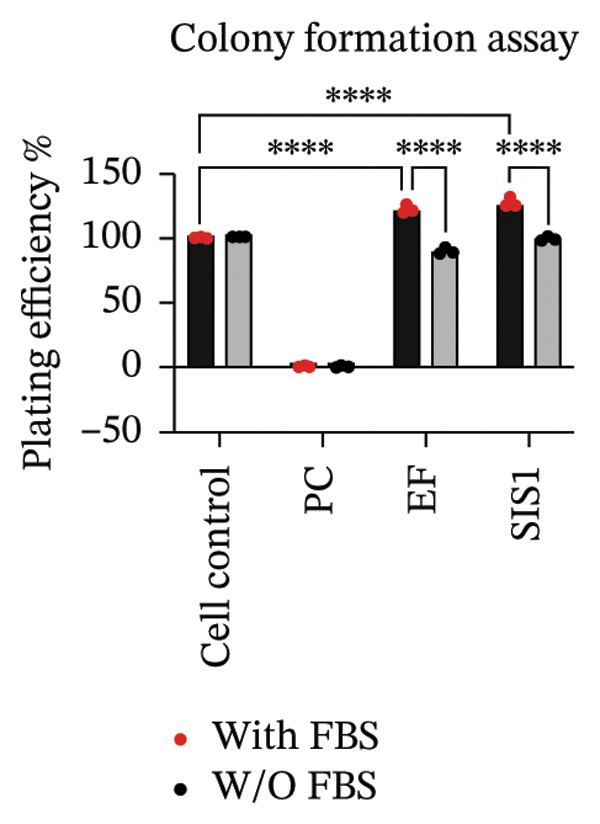
(d)

All four assays (MTT, XTT, NRU, and CFA) indicated that SIS1 and Endoform scaffolds were noncytotoxic; however, the metabolic activity readings were strongly influenced by the presence of FBS and assay types. In particular, the metabolic activity readings were significantly higher for the SIS1 scaffold in the MTT and XTT assays in the absence of FBS, indicating interference of serum with the test outcomes. Interestingly, this trend was not observed for the NRU assay. This is potentially because the NRU assay evaluates cell membrane integrity and viability, offering a more precise reflection of overall cell health [30]. In contrast, MTT and XTT assays measure mitochondrial activity, which can remain active in cells nearing death, potentially causing an overestimation of cell viability [11]. It has been previously demonstrated that different cytotoxicity tests can lead to varying cytocompatibility outcomes [22, 33, 54]. Cellular enzymes, proteins, lipids, and specific components found in cell media and serum are reported to reduce tetrazolium salts or solubilize formazan dye, potentially influencing the readings [12, 23, 24, 55]. MTT reduction is not confined to mitochondria but also occurs in the cytoplasm and at nonmitochondrial sites, including endosomal, lysosomal, and plasma membranes, and in certain cases may even proceed through noncellular mechanisms [38, 44, 56]. Another potential explanation for the higher metabolic activity in FBS‐free conditions can be due to the inherent presence of bioactive factors in SIS1 scaffold like proteins, GFs, or its degradation products that may augment cell metabolism in the absence of FBS. This is plausible as the SIS1 scaffold is derived from the ECM. The absence of FBS in the extraction medium may also enhance the elution of metabolism‐supporting bioactive factors from the scaffold into the culture medium. For example, it has previously been reported that serum in extraction medium can result in immediate coverage of a protein layer of fibronectin and albumin on material surfaces, thereby passivating the biomaterial [10, 14]. This could limit the release of the bioactive components from the scaffold into the test extraction medium, potentially explaining why increases in metabolic activity were only observed for the SIS1 extracts in the absence of FBS. When the SIS1 and Endoform materials were incubated with FBS‐containing extraction medium, a notable yellowish change in the medium’s color was observed, likely due to pH alterations [57]. This may arise from the interaction and adherence of FBS proteins with the test material, which would also deplete the test extraction medium of serum proteins with a pH shift.

A limitation of assays that rely solely on metabolic activity is their inability to distinguish between overall metabolic rate changes versus cell number or viability variations. Metabolic activity can vary significantly due to various factors, including cellular stress, nutrient deprivation, and/or inherent differences in metabolic rates between cell phenotypes [9, 26, 58]. To ensure a comprehensive cytotoxicity assessment, and to account for the potential influence of cell type‐specific effects, the impact of different cell types on cytotoxicity outcomes for the ISO 10993‐5 assays were evaluated.

### 3.2. Influence of Cell Type on Cytotoxicity Assessment of ECM Biomaterials Using ISO Recommended Assays

The ISO‐10993‐5 standard recommends immortalized cell lines for cytotoxicity assessment of biomaterials owing to their standardized nature, relative ease of use, and cost‐effectiveness. However, immortalized cell lines do not accurately replicate primary cell responses to biomaterials. For example, the L929 mouse fibroblast cell line, commonly used in the ISO 10993‐5 testing, exhibits known resistance to the toxicity of certain test agents, limiting applicability as a predictor of in vivo cytotoxicity [59]. BALB/c 3T3 or V79‐4 cells, which are also recommended in ISO 10993‐5 standard, are of mouse/hamster origin, which may not fully represent human cellular responses and sensitivity to toxins, potentially leading to inaccuracies in toxicity assessments. The cell membrane permeability of the reagents like MTT could also vary depending on different cell types [60]. MTT, XTT, and NRU assays using both primary cells and different cell lines were performed to investigate how cell type could influence cytotoxicity assessment of SIS1 scaffolds. The metabolic activity of primary HDF in response to the SIS1 scaffolds was assessed and compared to L929 and 3 T3 cell lines, along with a cancerous cell line (Caco‐2). In general, the SIS‐1 test extracts did not display any cytotoxic effects, with metabolic activity readings higher or comparable to cell controls in all conditions (Figures 3(a), 3(b), 3(c), 3(d), 3(e), and 3(f)). Interestingly, cell type‐specific responses were only observed in FBS‐free conditions. For example, L929 cells showed significantly higher metabolic activity compared to HDF and Caco‐2 cells in the MTT assay in FBS‐free conditions (Figures 3(b) and 3(d)). These results suggest that L929 cells can survive and maintain function in FBS‐free conditions more readily than primary cells. This robustness could limit the utility of L929 cells in studies aimed at replicating the more sensitive cellular responses to biomaterials observed in primary cells. It should be noted that significant increases in metabolic activity for the L929 cells were only observed in the MTT assay, highlighting the importance of employing multiple assay types and cell types before drawing conclusions from ISO‐10993‐5 recommended tests. Moreover, these cells may not reflect the actual cell types interacting with ECM scaffolds (e.g., stem cells, endothelial cells, and immune cells), leading to misleading toxicity profiles. Taken together, these results suggest it is advisable to employ a panel of cell types, including primary cells, when testing biomaterial cytotoxicity. The study results also demonstrate that ISO‐10993‐5 assays should be performed in the presence or absence of FBS test conditions to ensure accurate cytotoxicity assessment.

FIGURE 3Influence of different cell types (L929, 3T3, HDF, and Caco‐2 cells) on metabolic activity of SIS1 test extracts at different dilutions (100%‐12.5%) using: (a and b) MTT, (c and d) XTT, and (e) (f) NRU cytotoxicity tests compared between controls (cell control‐untreated cells growing on TCPS and positive control (PC; diluted SDS/SLS) and serum‐containing (with FBS) and serum‐free (without; W/O FBS) culture and extraction condition groups, respectively. Bar graphs represent *n* = 4 biological replicates, mean ± S.D.; ^∗^ *p* < 0.05, ^∗∗^ *p* < 0.01, ^∗∗∗^ *p* < 0.001, and ^*∗∗∗∗*^ *p* < 0.0001 versus cell control.

**Figure:**
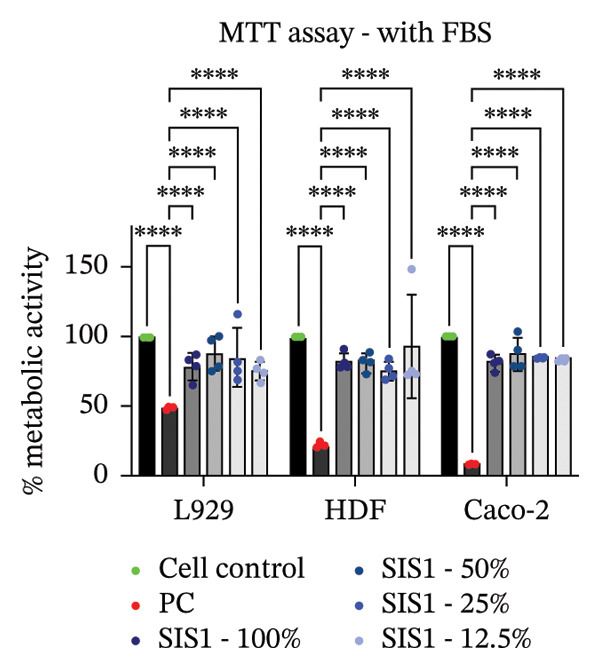
(a)

**Figure:**
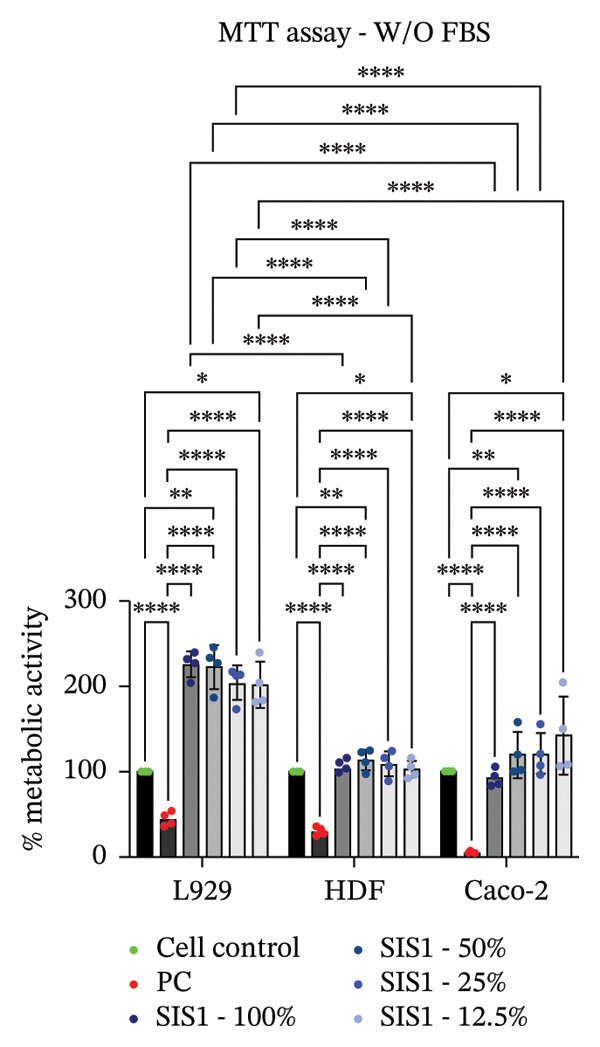
(b)

**Figure:**
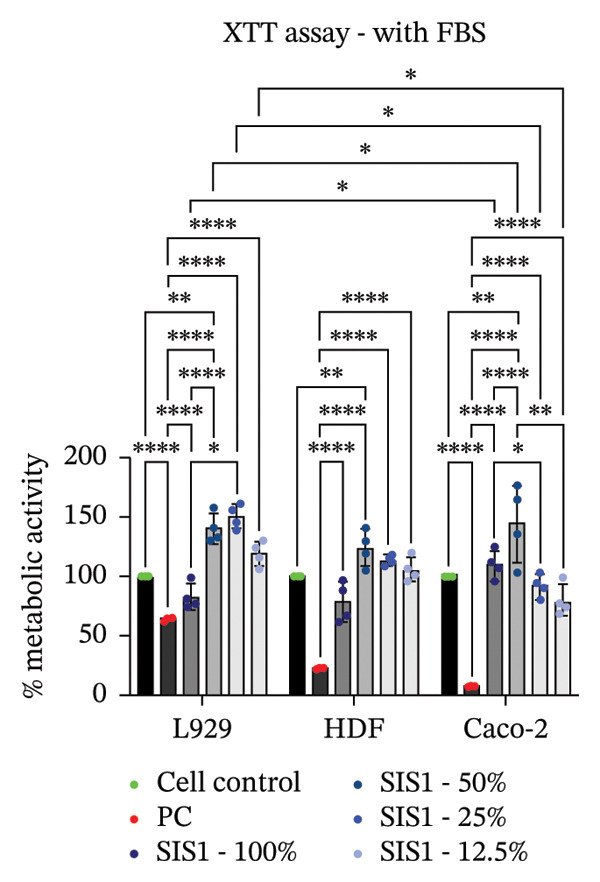
(c)

**Figure:**
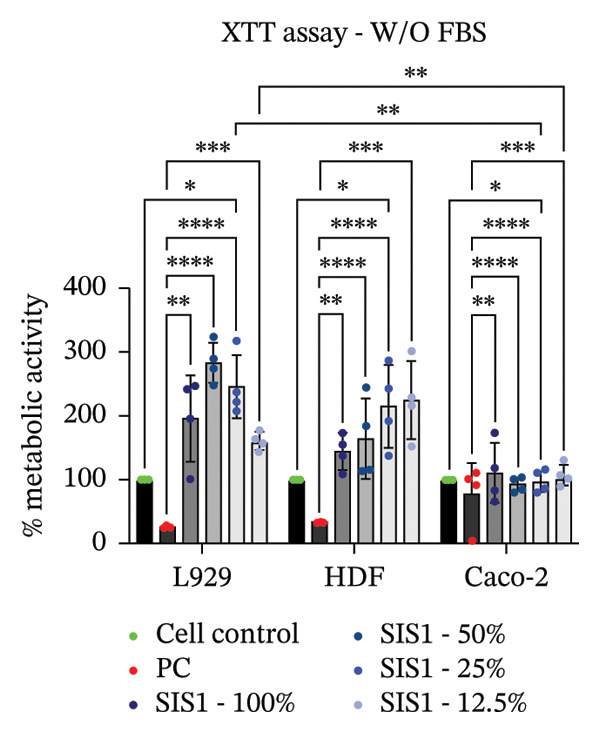
(d)

**Figure:**
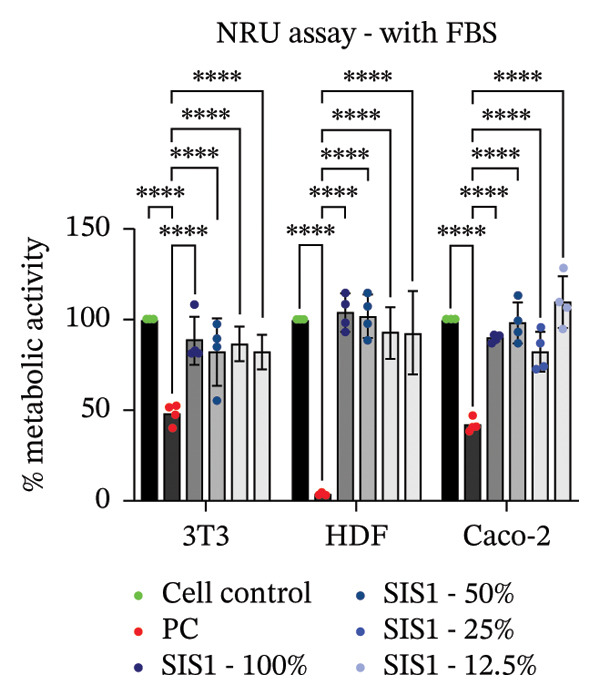
(e)

**Figure:**
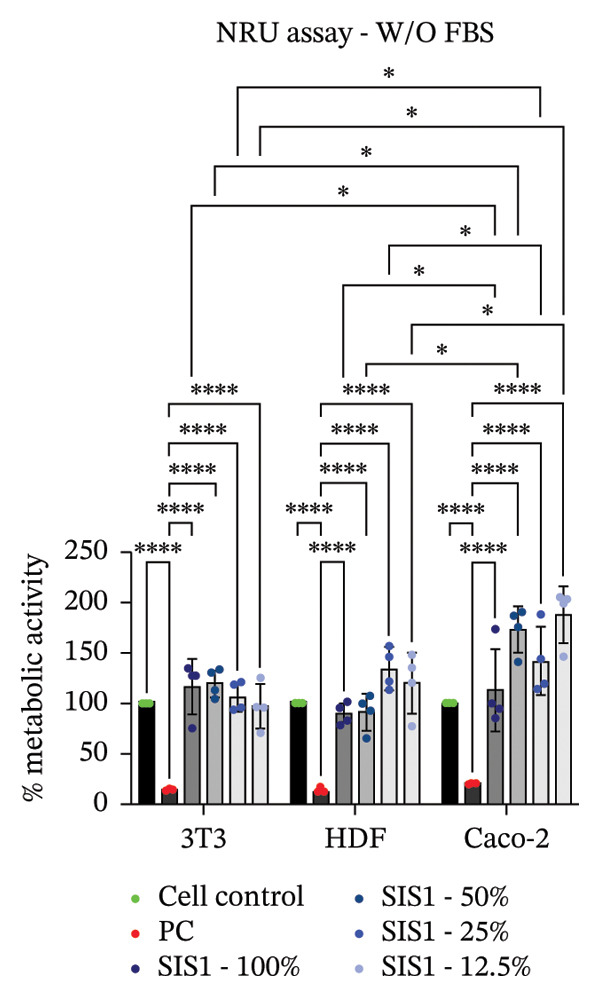
(f)

### 3.3. Influence of Cell‐Biomaterial Contact Type on Cytotoxicity Results

As per ISO 10993‐5, cytotoxicity evaluation is performed after biomaterial contact either directly, via direct contact or testing on extracts, or indirectly, through methods like agar or filter diffusion [3, 5]. Focusing on direct contact evaluation, the cytotoxicity of the SIS1 scaffolds was assessed using (i) exposure to scaffold extracts (test‐on‐extract) and (ii) direct contact with the scaffold material itself (Figures 4(a) and 4(b)). These assays were performed using L929 cells in accordance with ISO 10993‐5, and cellular responses were examined microscopically with particular emphasis on cell morphology and cytoplasmic integrity. Under the test‐on‐extract condition, SIS1 extracts at concentrations of 100%, 50%, 25%, and 12.5%, along with a 100% extract of Endoform, were evaluated. For direct contact testing, individual test specimens were positioned centrally within each culture vessel, covering approximately one‐10th of the cell monolayer surface, in accordance with ISO 10993‐5 guidelines. SpongeCol as a noncytotoxic biomaterial scaffold (NC) and toxic PC (0.1 mg/mL SDS/SLS) were employed for comparison along with the cell control. SpongeCol is a columnar interpenetrating porous collagen (bovine purified type‐I collagen) sponge that supports cellular attachment and proliferation (SpongeCol; Sigma, USA) [61]. Normal cell morphology was maintained with cells in contact with the NC biomaterial (grade 0), whereas the PC biomaterial showed grade 3 or 4 characteristics, with more than 70% of cells rounded or lysed and nearly complete destruction of the cell layers. Both the test‐on‐extract and direct test contact conditions demonstrated the noncytotoxic nature of the SIS1 scaffold, with no observable changes in cell morphology, lysis, or cell growth compared to the NC control (grade 0) (Figures 4(a) and 4(b)). No discernible differences were observed between the two test material contact conditions. A difference between these two contact conditions may be expected if the material releases cytotoxic leachables or if direct surface contact induces cell stress independent of extractable components. In the present study, no observable difference in cell morphology, lysis, or growth was detected between the test‐on‐extract and direct contact conditions. This suggests that the SIS1 test scaffold does not release cytotoxic soluble components at biologically relevant concentrations, and direct physical contact with the scaffold surface does not adversely affect cell viability or morphology.

FIGURE 4Influence of contact type on cytotoxicity assessment: (a and b) comparison of microscopic evaluation of direct contact assay using L929 cells after 24 h of culture between contact types (test extracts (different dilutions of SIS1 test extracts—100%, 50%, 25%, and 12.5% and Endoform [EF]—100%) and direct test contact) and controls (cell control‐untreated L929 cells on TCPS), negative control (NC; SpongeCol; Sigma), and positive control (PC; diluted SDS/SLS), respectively; scale bar = 200 μm and (c–e) comparison of cell viability using MTT cytotoxicity test using L929, HDF, and Caco‐2 cell types after 24 h of culture with 100% SIS1 test extract between cell control and with FBS and W/O FBS culture and extraction condition groups, respectively. Data graphs represent *n* = 4 biological replicates, mean ± S.D.; ^∗^ *p* < 0.05 and ^∗∗^ *p* < 0.01 versus cell controls.

**Figure:**
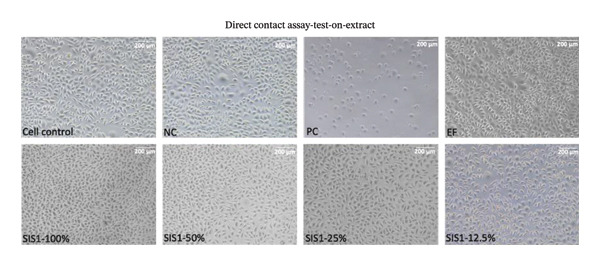
(a)

**Figure:**
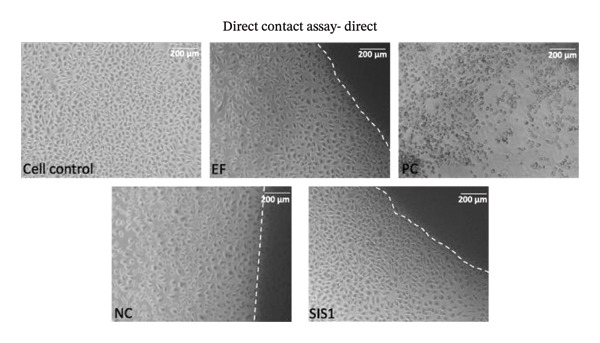
(b)

**Figure:**
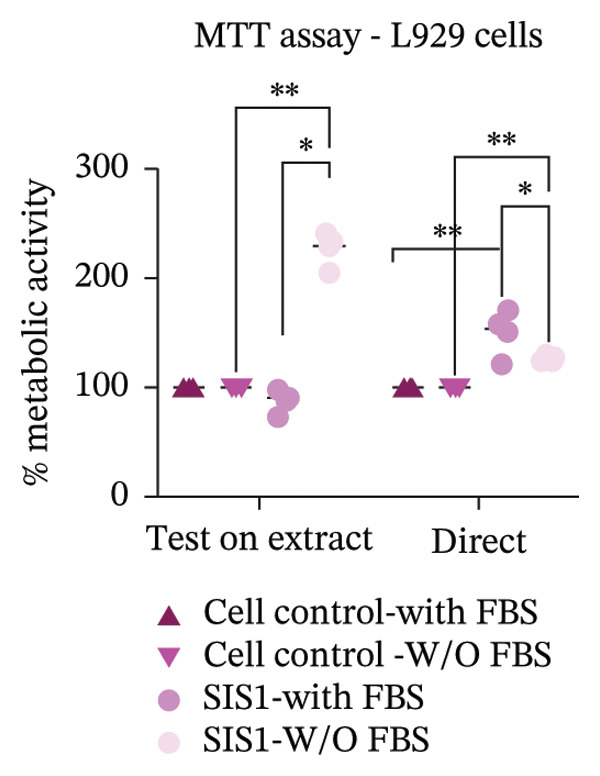
(c)

**Figure:**
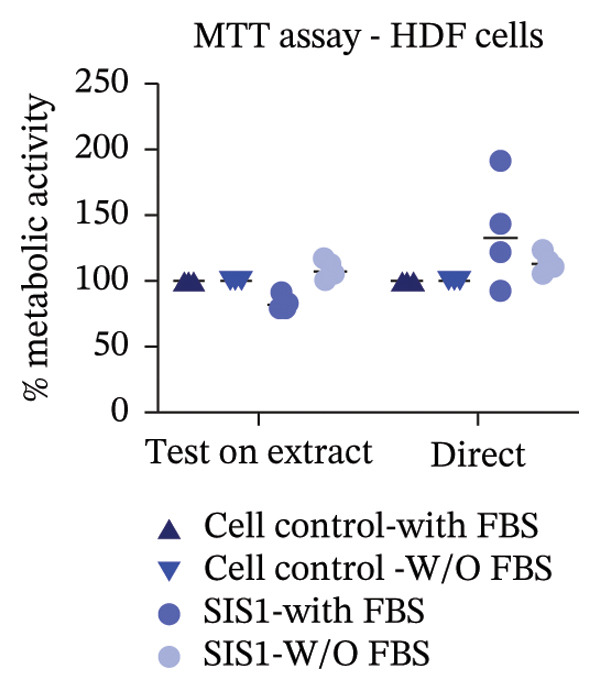
(d)

**Figure:**
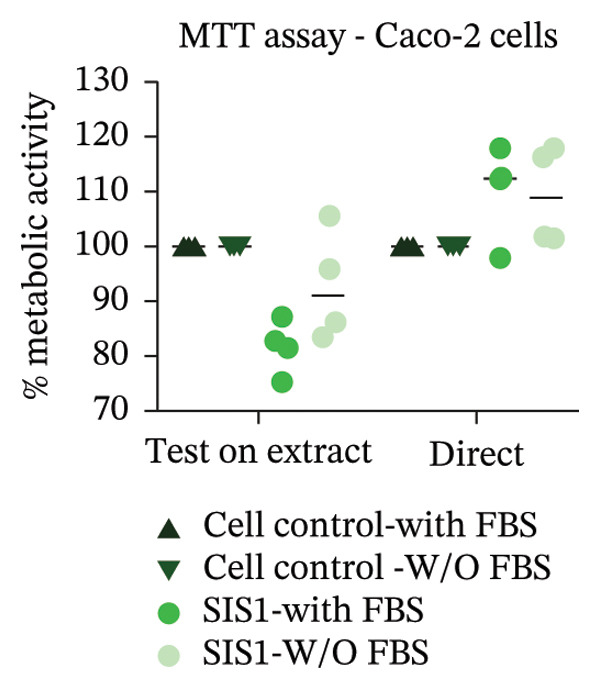
(e)

Next, the MTT assay was used to compare metabolic activity for cells exposed to SIS1 test extracts (test‐on‐extract contact) and cells seeded directly (direct contact) on the SIS1 test material using L929, HDF, and Caco‐2 cells (Figures 4(c), 4(d), and 4(e)). Seeding cells directly onto the test scaffold evaluates surface‐mediated and physical interactions which can influence cell adhesion, morphology, and growth, whereas exposure to test scaffold extracts assesses soluble leachable toxicity. In vivo, cells interact with ECM in a complex, dynamic manner that influences their function and viability. The architecture of a scaffold influences nutrient diffusion, oxygen availability, and waste removal, while its stiffness affects mechano‐transduction pathways, and surface topography, including roughness and micro/nano‐scale patterns, modulates cell attachment and cytoskeletal organization, thereby impacting overall cell survival [62, 63]. The absence of differences between these contact conditions indicates that the scaffold is both chemically noncytotoxic and supportive of normal cell–material interactions. In the absence of FBS, significantly higher metabolic activity of L929 cells was observed in the SIS1 test‐on‐extract conditions compared to nontreated cell controls and with FBS (Figure 4(c)). This agrees with earlier results suggesting bioactive components in the SIS1 test extracts can promote L929 metabolic activity. When the MTT assay was performed using primary HDF cells, no significant differences were observed between the contact types in either ± FBS groups (Figure 4(d)). Similarly, no significant differences were observed between the test conditions when using Caco‐2 cells (Figure 4(e)). The enhanced metabolic activity of the L929 cells may be due to cell‐specific interactions with the ECM scaffolds (e.g., ligand type and integrin expression) or cell‐type‐specific metabolic requirements. ECM‐based biomaterials can influence cellular behavior by affecting adhesion, proliferation, and metabolic activity [42, 49]. Cells cultured on or within ECM scaffolds may adopt a different metabolic state compared to standard 2D cultures, making direct comparisons with traditional metabolic assays unreliable [43, 49]. Standard metabolic assays and specifications may not fully capture these interactions, leading to discrepancies between in vitro and physiological conditions.

### 3.4. Incorporation of Staining Methods for Cytotoxicity Evaluation

An alternative approach to relying solely on chemical readouts by colorimetric metabolic assays or colony counting is to assess biological outcomes such as cell adhesion, proliferation, DNA synthesis, cell cycle progression, senescence, and indicators of apoptosis or necrosis by measuring specific marker expressions over time, providing a more physiologically relevant evaluation. Live/dead staining is a simple dual fluorescent staining technique that enables simultaneous differentiation between live and dead cells by evaluating intracellular esterase activity and membrane integrity using calcein AM and EthD‐1 dyes, allowing a clear distinction between the two populations. This provides more precise information on cellular responses compared to population‐average assessments of metabolic activity. Live/dead staining was compared between fibroblasts of mouse origin (L929 cells) and human origin (HDF) cultured on the test SIS1 material under direct contact and test‐on‐extract conditions, relative to cell controls (Figures 5(a) and 5(c)). Both cell types were viable on both direct SIS1 test and test‐on‐extract contact groups after 24 h and 72 h, indicating the noncytotoxic nature of the SIS1 scaffold (Figures 5(a) and 5(c)). Cell morphology was more spindle‐like in the direct SIS1 scaffold contact group, potentially due to the fibrous and porous nature of the 3D scaffold compared to 2D culture conditions (Figures 5(a) and 5(c)). Quantification of cell viability using FIJI image analysis software demonstrated cell viability in the range of 90%–95% based on the total cell count in the sample for both test conditions at 24 h and 72 h in the FBS‐containing groups and cell types (Figures 5(b) and 5(d)). Under FBS‐free test conditions, both cell types exhibited slightly lower cell numbers in the direct contact and test‐on‐extract groups compared to the cell control. Although this difference is not reflected in the percentage viability data, it is evident in the qualitative imaging results (Figures 5(a) and 5(b)). L929 cells exhibited a significant increase in viability at 24 h in the direct contact group, whereas by 72 h, cell viability remained unchanged in both contact groups compared to their respective controls. In the absence of FBS, L929 cells showed a significant increase in viability at 24 h in the test‐on‐extract group, but no significant difference was observed at 72 h in either contact condition. In contrast, HDF cells consistently maintained significantly higher viability at both 24 h and 72 h under FBS‐supplemented conditions, regardless of whether they were in direct contact or test‐on‐extract groups. However, in the absence of FBS, HDF cells only showed a significant increase in viability at 24 h, with minimal differences observed at 72 h (Figures 5(c) and 5(d)). Notably, live/dead staining results did not replicate the increases observed with MTT and XTT assays. These differences likely arise because metabolic assays reflect overall enzymatic activity, which may transiently elevate even in stressed cells, whereas live/dead staining directly evaluates cell membrane integrity and esterase activity, offering a more accurate measure of true cell survival.

FIGURE 5Cell viability analysis using live‐dead staining: (a and b) microscopic images of multiple wells showing calcein AM‐ethidium homodimer‐1 (EthD‐1) staining depicting live L929/human dermal fibroblast (HDF) cells (green) and dead L929/HDF cells (red) on the test SIS1 directly and on test‐on‐extract (contact types), respectively, of *N* = 3 biological replicates; scale bar = 500 μm and (c and d) their quantitative analysis of live and dead cells (%) using FIJI image analysis software compared between two culture time points (24 h and 72 h), cell types (L929 and HDF) and contact types in serum‐containing (with FBS) and serum‐free (without W/O FBS) culture and extraction conditions, respectively. Data graphs represent *n* = 9 measurements, mean ± S.D.; ^∗^ *p* < 0.05, ^∗∗^ *p* < 0.01, ^∗∗∗^ *p* < 0.001, and ^*∗∗∗∗*^ *p* < 0.0001.

**Figure:**
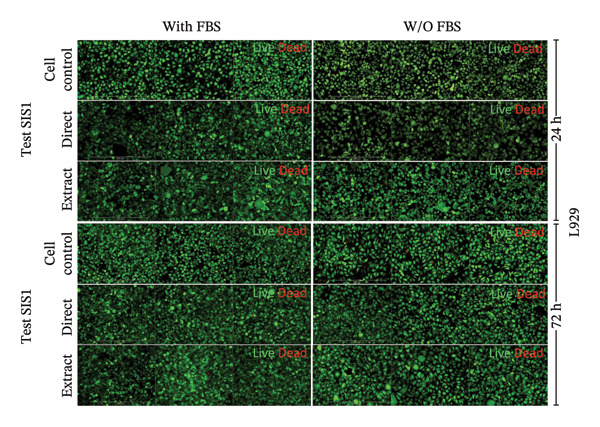
(a)

**Figure:**
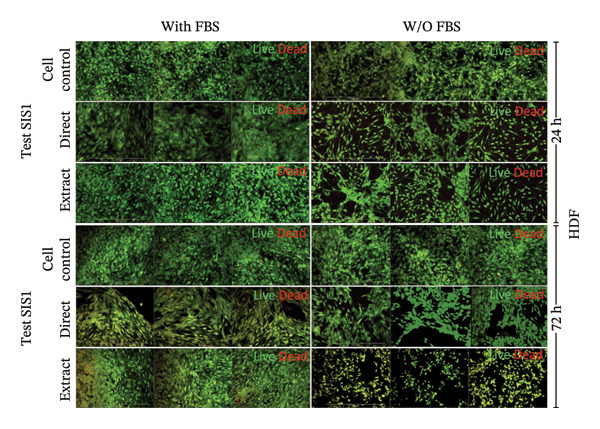
(b)

**Figure:**
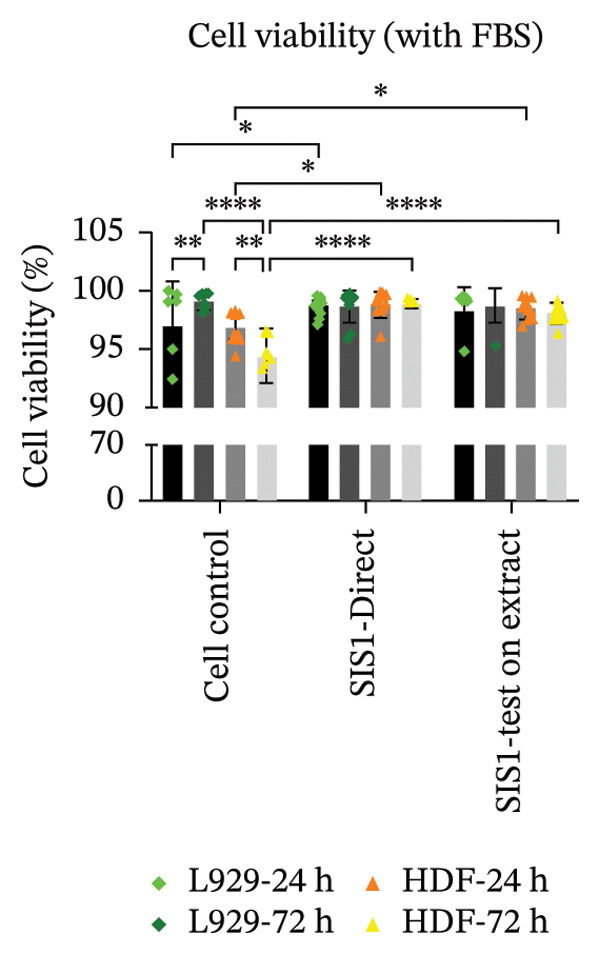
(c)

**Figure:**
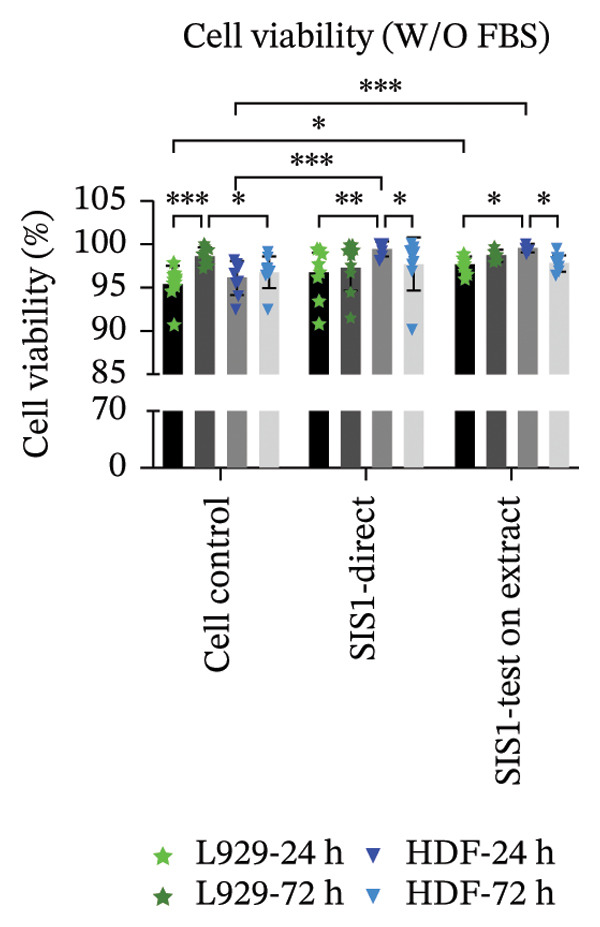
(d)

Next, the alterations in actin cytoskeleton organization in both direct contact and test‐on‐extract conditions were examined using rhodamine‐phalloidin actin staining. F‐actin staining enables the assessment of cell spreading, adhesion, and cytoskeletal organization, providing insights into material‐induced effects such as cellular stress, apoptosis, or cytotoxicity [64, 65]. A comparative analysis was conducted between mouse‐derived (L929) and human‐derived (HDF) fibroblast cells. Actin staining revealed robust cell spreading and well‐developed cytoskeletons in direct scaffold contact and the test‐on‐extract conditions, further confirming the cytocompatibility of the SIS1 scaffold in both cell types (Figures 6(a) and 6(c)). The fluorescence intensity values were quantified by measuring the CTCF, which corrects for background fluorescence to quantify the fluorescence intensity of individual cells or specific regions of interest within cells. Both the cell types did not show any significant increase in CTCF values in the presence of FBS in both direct contact and test‐on‐extract groups. However, L929 cells showed reduction of CTCF values in test‐on‐extract group at 24 h and HDF cells showed reduction of CTCF values between the contact types (Figure 6(b)). Under FBS‐free conditions, L929 cells exhibited reduced actin CTCF values in the test‐on‐extract group at 24 h and in the direct contact group at 72 h. A time‐dependent decline in actin CTCF values was also observed in L929 cells when comparing 24 h–72 h across both contact types. Similarly, HDF cells demonstrated reduced actin CTCF values across both contact groups and time points when compared to their respective control cells (Figure 6(d)). Interestingly, in the absence of FBS, actin immunostaining did not reveal a significant increase in CTCF under either direct contact or test‐on‐extract conditions, in contrast to the metabolic activity results obtained from MTT and XTT assays. This discrepancy may suggest that while cellular metabolism remained detectable, cytoskeletal organization and attachment were slightly compromised under serum‐free conditions.

FIGURE 6Cell adhesion and spreading assessment using actin immunostaining: (a and b) microscopic images displaying multiple wells stained with rhodamine phalloidin for actin (red; cell cytoskeleton) and DAPI/Hoechst (blue; nuclei), illustrating L929 and human dermal fibroblast (HDF) cells cultured on the SIS1 surface directly and on test‐on‐extract (contact types) in serum‐containing (with FBS) and serum‐free (without W/O FBS) culture and extraction conditions compared between two culture time points (24 h and 72 h), respectively, across three biological replicates (*N* = 3); scale bar = 500 μm and (c and d) their quantitative analysis of corrected total cell fluorescence (CTCF) using FIJI image analysis software compared between culture time points, cell types, contact types, and serum‐containing and serum‐free culture and extraction conditions, respectively. Bar graphs represent *n* = 6–9 measurements, mean ± S.D.; ^∗^ *p* < 0.05, ^∗∗^ *p* < 0.01, ^∗∗∗^ *p* < 0.001, and ^∗∗∗∗^ *p* < 0.0001.

**Figure:**
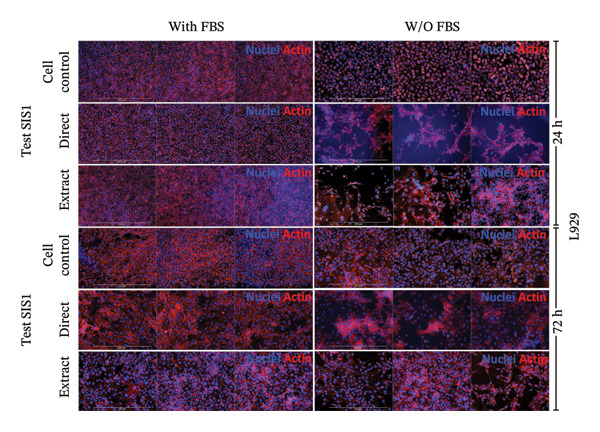
(a)

**Figure:**
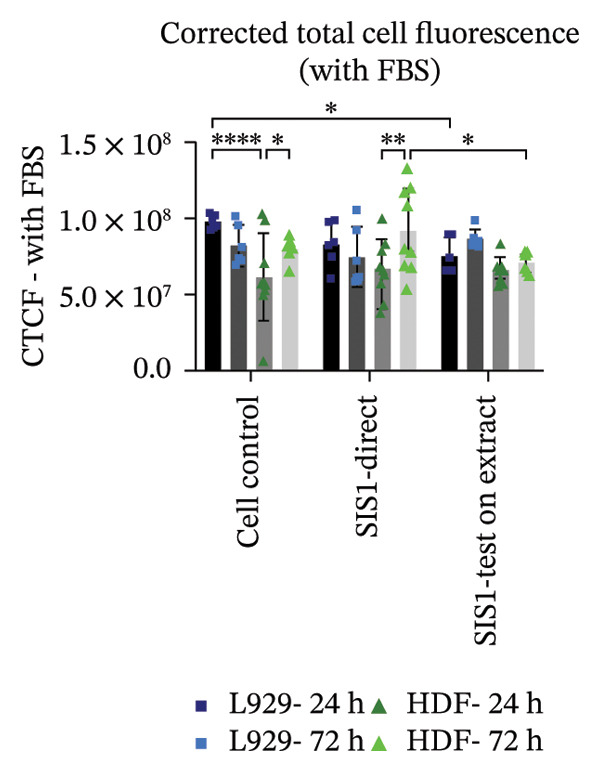
(b)

**Figure:**
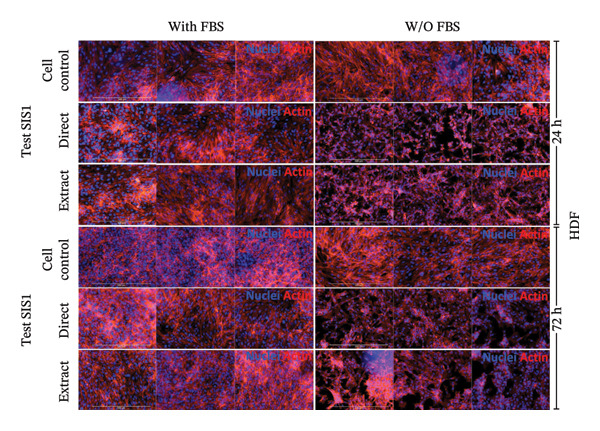
(c)

**Figure:**
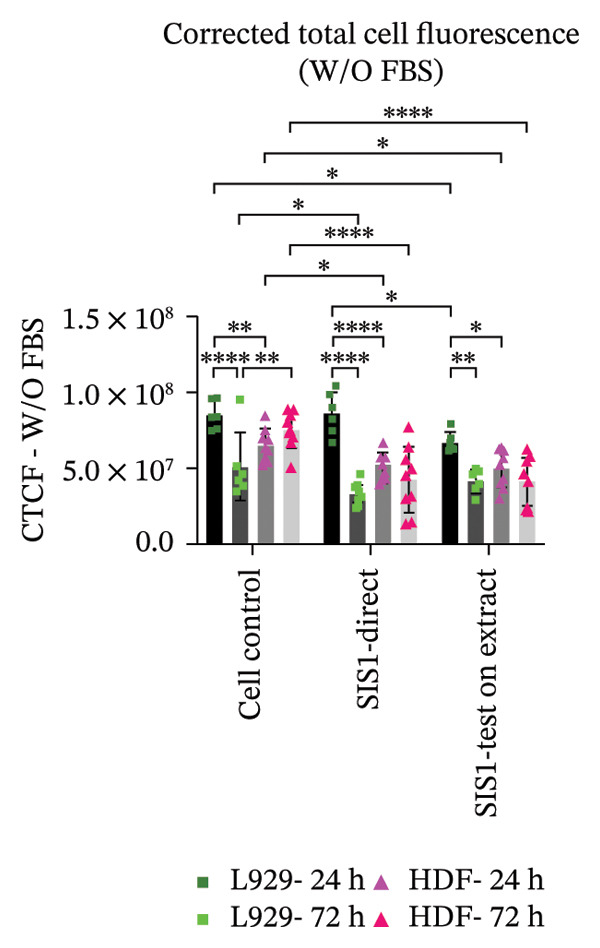
(d)

Another parameter that can provide information on the cytotoxicity of ECM biomaterials is evaluating proliferation rates for cells seeded on the biomaterial. Lower cell proliferation may indicate biomaterial cytotoxic effects, while unaffected or higher proliferation suggests compatibility with cell growth and viability. Ki67 staining was employed to measure the proliferation of cells in contact with the SIS1 scaffold. Two contact methods were employed, direct contact and indirect contact via a scaffold extract, and proliferation was assessed at 24 and 72 h between mouse (L929) and human‐derived (HDF) fibroblast cells (Figures 7(a), 7(b), 7(c)). Ki67 positivity was comparable between direct contact and test‐on‐extract conditions in the FBS group and was similar to that observed in the serum (FBS)‐containing cell controls (Figures 7(a) and 7(b)). Under serum‐free conditions, Ki67 positivity was reduced relative to controls across both contact types and cell types, with a significant difference between contact types at 72 h in HDF cells (Figures 7(a) and 7(c)). Interestingly, although cell counts declined in the absence of serum, this reduction was not proportionally reflected in Ki67 positivity, suggesting that a subset of cells retained proliferative capacity despite overall stress. The results also highlight the importance of incorporating additional time points in experimental readouts to capture cellular activity beyond the 24 h window recommended for most ISO 10993‐5 assays. The results suggests that bioactive factors released from the ECM scaffold could have modest impacts on cell proliferation. Finally, annexin V‐PE staining was utilized to detect apoptosis than L929 and HDF cells exposed to the SIS1 scaffold in direct and test‐on‐extract conditions after 24 h culture (Figures 8(a), 8(b), and 8(c)). Interestingly, no positive staining was observed in any groups in L929 cells, whereas the HDF cells showed apoptosis ranging from 0.6% to 9.3% (less than 10%) in both serum‐containing and serum‐free groups (Figures 8(a), 8(b), and 8(c)). This highlights that sensitivity to cytotoxic stimuli may vary between cell lines with greater sensitivity of HDF cells to L929 cells, demonstrating that human‐derived cell lines may be more effective in detecting subtle changes in cytotoxicity.

FIGURE 7Cell proliferation analysis using Ki67 immunostaining: (a and b) microscopic images of proliferation marker Ki67 (green)‐DAPI (nuclei; blue) immunostaining compared between L929 and human dermal fibroblast (HDF) cells on the test SIS1 directly and on test‐on‐extract, respectively of *N* = 3 biological replicates; scale bar = 100 μm and (c and d) their quantitative analysis of Ki67 positive cells with *n* = 9 measurements using FIJI image analysis software compared between two culture time points (24 h and 72 h), cell types, contact types, and cell control in serum‐containing (with FBS) and serum‐free (without W/O FBS) culture and extraction conditions, respectively. Data graphs represent *n* = 9 measurements, mean ± S.D.; ^∗^ *p* < 0.05, ^∗∗^ *p* < 0.01, and ^∗∗∗^ *p* < 0.001.

**Figure:**
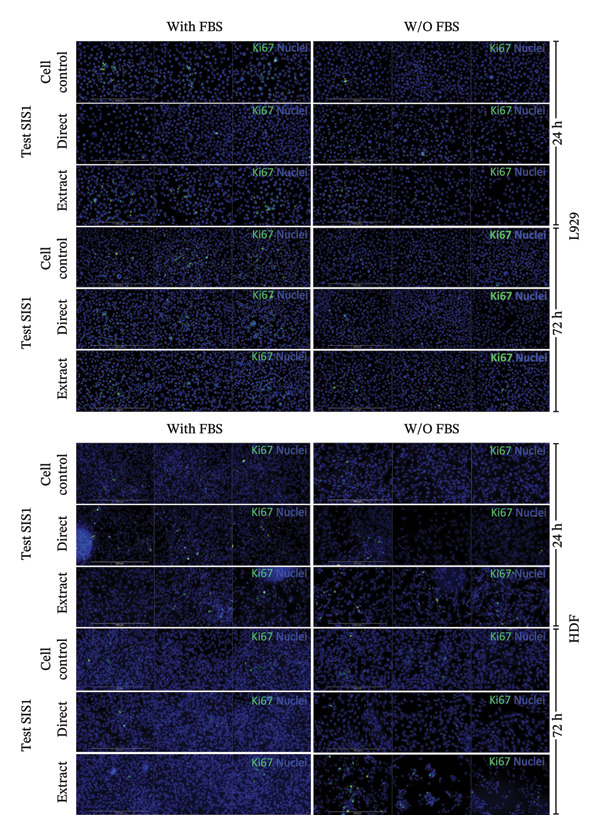
(a)

**Figure:**
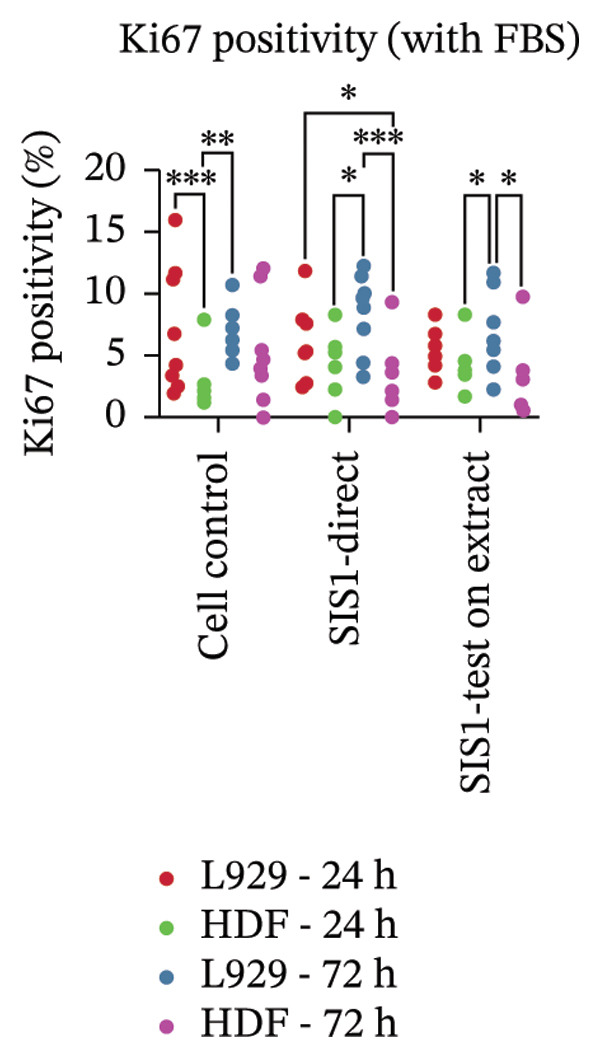
(b)

**Figure:**
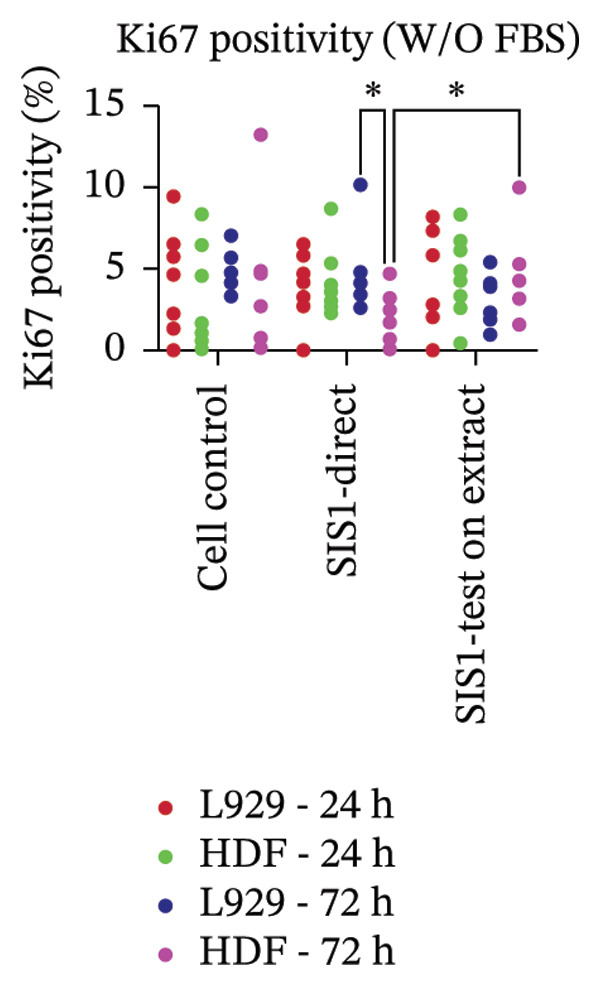
(c)

FIGURE 8Microscopic analysis of the binding of PE‐conjugated Annexin V to apoptotic L929 and human dermal fibroblast (HDF) cells after treating them with the test sample directly and on test‐on‐extract: (a) Microscopic images showing panel of multiple wells stained with apoptotic marker annexin V‐PE (red) and Hoechst (nuclei; blue) after 24 h of culture in the presence/absence of serum (FBS) in extraction and cell culture conditions, respectively of *N* = 3 biological replicates; scale bar = 500 μm, and (b) and (c) quantitative analysis of annexin V‐PE positive cells using FIJI image analysis software compared between contact types and cell types in serum‐containing (with FBS) and serum‐free (without W/O FBS) culture and extraction conditions, respectively. Data graphs represent *n* = 6–9 measurements, mean ± S.D.; ^∗∗∗∗^ *p* < 0.0001.

**Figure:**
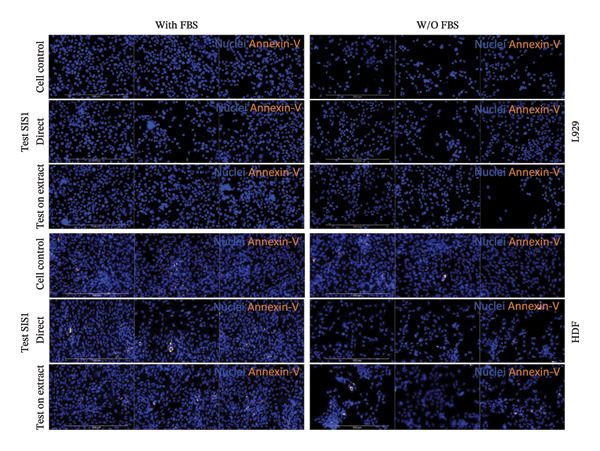
(a)

**Figure:**
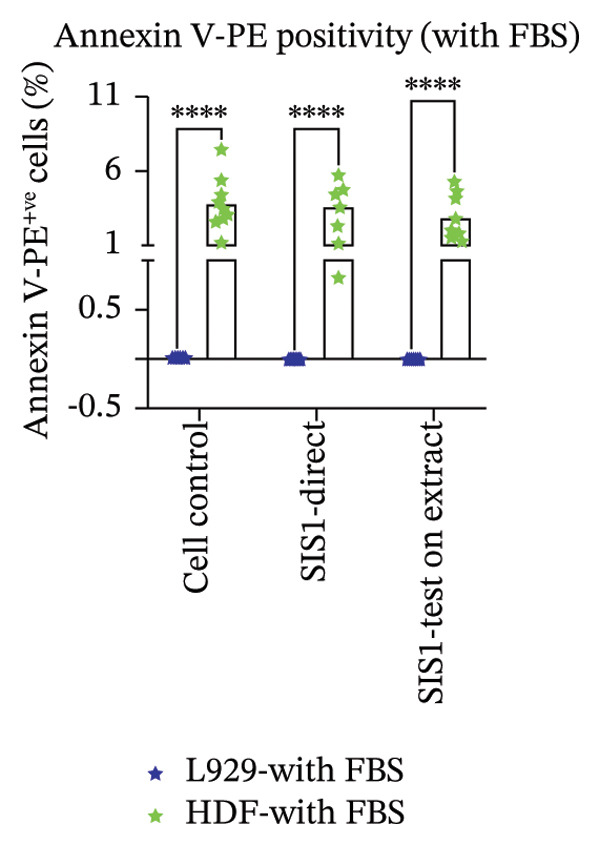
(b)

**Figure:**
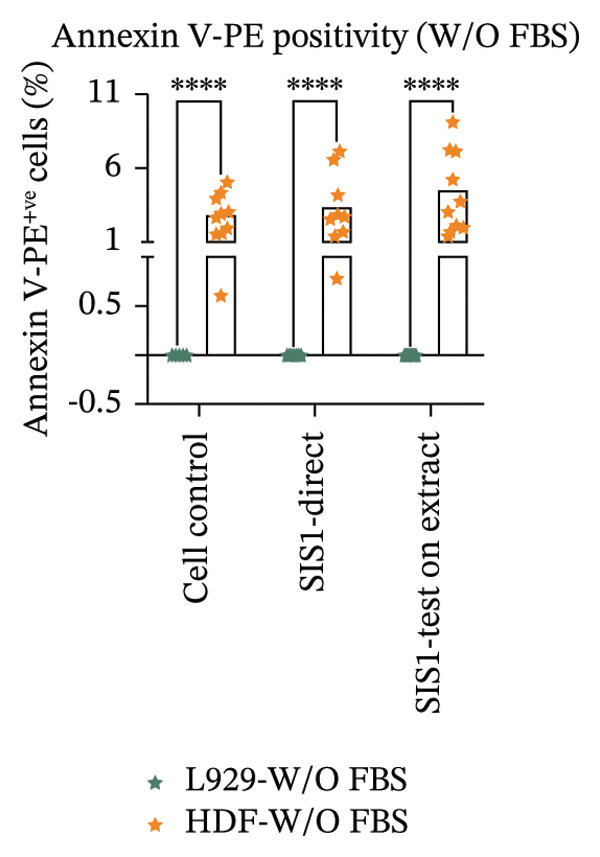
(c)

The results highlight the limitations of relying solely on single metabolic activity assays, such as those recommended by ISO 10993‐5 (MTT, XTT, NRU, or CFA), for assessing the cytotoxicity of ECM‐based biomaterials. Specifically, despite increased metabolic activity indicated by MTT and XTT assays in FBS‐free extract conditions, live/dead and actin staining revealed no corresponding increase in cell viability. While modest increases in cell proliferation were observed for the scaffold extracts in the absence of FBS, this was significantly lower than the 100%–150% increases in metabolic activity. Additionally, the NRU assay, which uses a different metabolic indicator, did not indicate increases in metabolic activity. These results suggest that the enhanced metabolic readings were due to the interference of specific scaffold components with assay reagents rather than due to the inherent mitogenic or bioactive factors present in the scaffolds. This highlights the limitations of using a single assay for monitoring cytotoxicity and indicates that multimodal characterization techniques should be employed. These findings further emphasize the need to account for various parameters when designing test specifications, including the choice of cell type, the type of material‐cell contact, and the potential interference of FBS in the case of ECM‐based biomaterials. The validation of a staining assay for potential inclusion in ISO‐standard cytotoxicity testing requires a thorough evaluation of key parameters: specificity, sensitivity, accuracy, precision, reproducibility, and robustness. Specificity is assessed by comparing results to appropriate controls to ensure selective detection of target cell states (e.g., viability, apoptosis, proliferation) without interference or cross‐reactivity. Sensitivity is tested by exposing cells to a range of concentrations of a known cytotoxic agent, confirming the assay’s ability to detect subtle changes in cellular responses. Accuracy is determined by comparing the assay results with those obtained from established ISO‐approved methods, such as MTT, XTT, or NRU. Precision is evaluated by performing the assay over multiple days to assess consistency in results. Reproducibility is confirmed by conducting the assay across different operators and laboratories. Robustness is tested by introducing minor procedural variations (e.g., ±10% in incubation time or reagent concentration) and verifying that results remain within an acceptable variability threshold, typically less than 10%. In this study, we evaluated the specificity, accuracy, and precision of several recommended staining methods (live/dead, actin cytoskeleton, proliferation via Ki67, and apoptosis via annexin V).

Assessment of cell membrane integrity (live/dead), cell attachment (actin), proliferation (Ki67), and apoptosis (annexin V) offers a more comprehensive evaluation of cellular responses to ECM‐based biomaterials than standard ISO 10993‐5 assays (MTT, XTT, NRU, and CFA). Although numerous studies have emphasized the limitations of the assays outlined in the ISO 10993‐5 standard as highlighted in Table 2 [9–11, 13, 14], this study has comprehensively evaluated the ISO 10993‐5 recommended assays in the context of ECM‐based scaffolds. Since its initial release in 1992, the ISO 10993‐5 standard has undergone two revisions, with the most recent update in 2009 [9]. The guidelines provided in ISO 10993‐5 lack sufficient specificity explicitly to ensure reliable and comparable results across different laboratories and biomaterials to ensure consistent and comparable results for the same medical device [9]. This study reinforces the importance of contextualizing the ISO 10993‐5 standard to incorporate better specifications and assays that assess a wider spectrum of cellular responses, such as those utilized and demonstrated in this study. Considering factors such as emerging classes of biomaterials, extraction conditions, contact or cell types, advanced methodologies, and biomarkers, as well as potential sources of interference, such as FBS, will improve the precision and robustness of in vitro cytotoxicity testing for biomaterials.

## 4. Conclusion

The study demonstrates that the ISO 10993‐5 standard for biomaterial cytotoxicity testing, while valuable, could be improved to enable more comprehensive characterization of ECM‐based biomaterials. The study systematically evaluated the assays recommended by the ISO 10993‐5 standard, focusing on critical variables, including cell type, type of contact (either with test extracts or the material itself), and media components (± serum), showcasing the drawbacks associated with the assays when used alone. Significant variability in cell response was observed across different colorimetric assays (MTT, XTT, NRU, and CFA), highlighting the potential for reagents/serum proteins to interfere with these assays. Furthermore, the findings underscore the importance of using primary/human cell lines relevant to the intended application, in addition to the standardized cell lines recommended by ISO 10993‐5. To achieve a more comprehensive evaluation of ECM‐based biomaterial cytotoxicity, the study suggests combining ISO‐recommended colorimetric tests with assays that provide more detailed insights into cell behavior, including techniques such as live/dead staining, cytoskeletal actin analysis, proliferation (Ki67), and apoptosis markers (annexin V staining). These improvements have the potential to streamline preclinical evaluations by providing a more accurate and reliable assessment of biomaterial cytotoxicity, ultimately reducing time and costs associated with biomaterial development.

## Funding

This publication has emanated from research funded by the Research Ireland, co‐funded grant under the European Regional Development Fund under Grant Number 13/RC/2073_P2, and Cook Biotech (now Evergen, USA; Project ID: TP1‐AD‐01).

## Disclosure

Pictorial illustrations used in graphical abstract, Table 1 and Figure 1 are created using BioRender.com.

## Conflicts of Interest

The authors declare that the research was in part funded by Cook Biotech (now Evergen, USA) through Research Ireland CÚRAM industry co‐funding model.

## Supporting Information

Additional supporting information can be found online in the Supporting Information section.

## Supporting information


**Supporting Information** The supporting document file contains the graphical abstract of this study. The graphical abstract illustrates the assessment of cellular responses to biomaterials using ISO 10993‐5 standard cytocompatibility evaluation methods. Cells are exposed to the biomaterial through three experimental approaches: test extract‐based testing, indirect contact, and direct contact or encapsulation within the scaffold matrix. Following biomaterial interaction, cells may exhibit a range of physiological states, including viable, stressed, apoptotic, or necrotic conditions. These responses are associated with characteristic morphological and membrane alterations, such as membrane perturbation, cell shrinkage, apoptotic body formation, cellular swelling, and membrane rupture. The observed cellular changes are subsequently analyzed to evaluate the cytocompatibility and cytotoxic potential of the biomaterial.

## Data Availability

All data are presented in the manuscript. The datasets supporting the findings of this study are openly available in Zenodo at https://doi.org/10.5281/zenodo.18495431.
